# Experimental studies of the influence of mobile fan positioning parameters on the ability to transport the air stream into the door opening

**DOI:** 10.1038/s41598-023-42147-5

**Published:** 2023-09-11

**Authors:** Piotr Kaczmarzyk, Łukasz Warguła, Paweł Janik

**Affiliations:** 1 Science and Research Centre for Fire Protection, National Research Institute, 05-420 Józefów, Poland; 2 Institute of Machine Design, Faculty of Mechanical Engineering, Poznań University of Technology, 60-965 Poznań, Poland

**Keywords:** Energy science and technology, Engineering

## Abstract

The article aims to determine the influence of fan positioning parameters, i.e., its distance from a door opening (1–7 m) and the angle of inclination of the impeller axis in relation to the ground (0°–18°) on the amount of air flow pumped through a door opening. The experiment was carried out using a mock-up simulating a door opening, on which a measurement plane was located, without the cubic capacity (building structure) behind the door opening. The volumetric air flow stream was determined based on measuring (at 50 measuring points) the velocity of the air stream blown onto the surface of the door opening mock-up. Four commercial positive pressure ventilators, commonly used in rescue operations, with a power of 0.6–6.3 kW were tested. The tests showed that the value of the air flow stream at the most favourable setting (distance in the range of 3–5 m and the angle of the impeller axis to the ground in the range of 5°–12.2°) is included in the range of 18,304 ± 2460 m^3^/h to about 45,189 ± 4619 m^3^/h. Such settings cause the air stream to be aimed at the central area of the door opening. Imprecise mobile fan arrangement may reduce the flow rate from 41 to 76% in relation to the most favourable results.

## Introduction

Internal fires of building structures pose a great threat to the users^1^. During fires, the greatest threat is the emission of toxic products from thermal decomposition and combustion^2^. The emission of such substances is related to the combustion of plastics or impregnated materials^3–5^.

To combat the risk of smoke during internal fires, firefighters use mobile fans taking into account various mechanical ventilation techniques, e.g., positive pressure ventilation (PPV) or negative pressure ventilation^6^. The literature on the subject also indicates other possibilities of using these devices, respectively: supporting activities related to the elimination of fire hazards of free-standing objects (e.g. cars or garbage containers), ventilation of hard-to-reach places (where people stay)—with the use of electric fans^6^. Referring to devices (including positive pressure ventilators) used in rescue operations, it is indicated that they should be characterized by high operational reliability, efficiency, and functionality and should have little impact on the user^6,7^. In many countries, devices used in rescue operations are subject to special testing procedures^8^. Referring to mobile fans, so far, no requirement has been introduced in Poland to conduct tests verifying the effectiveness of this type of device. For positive pressure ventilators, there are many methodologies^9,10^ used to test the volume flow rate. However, it should be emphasized that, depending on which methodology is selected for the test, the obtained results may differ significantly and their incorrect interpretation may mislead firefighters in terms of their actual suitability for rescue operations. Regarding the fans used by the rescuers, the conditions under which the tests are carried out are very important—open flow. The values of the volumetric flow test results will vary depending on the test conditions. As shown by the analyses conducted by Kaczmarzyk et al. in 2022, the open-air flow measurement method better reflects the actual conditions of the device use, and the driving power demand, depending on the test method, may vary from 3.2 to 4.5%^7,8^. Additionally, Fritsche et al. in 2018 indicated that the manufacturers’ declared volumetric airflow rate values for mobile fans may differ from actual ones due to the lack of a standardized methodology for testing this type of equipment^11^.

Air flow value during open flow tests may be influenced by the distance of the fan from the inlet duct and the position of the impeller in relation to the ground. Lougheed et al., in 2002, analyzed different distances of the fan positioning concerning the door opening (1.2 m, 1.8 m and 2.8 m). During the tests, it was shown that the highest air flow was obtained when the fan was located 1.2 m from the door plane^12^. On the other hand, in 2017 Panindre et al. carried out research on the analysis of the impact of the size of the inlet on the efficiency of positive pressure ventilation PPV (PPV is a technique based on ventilating the building through the inlet with a movable fan placed in front of the inlet)^13^. The research showed that the flow rate can increase thanks to the installation of a mobile smoke curtain in the upper area of the door frame^13^. In the same year, the authors, using a Fire Dynamics Simulator (FDS 5.0), conducted research on the impact of the rescue capabilities of fans depending on the structural layout of a building, wind conditions and the arrangement of the fans, demonstrating that the effectiveness of PPV decreases with increasing wind speed^14^. Mobile positive pressure fans are characterized not only by air flow but also by the shape of the air stream (the surface of the effective distribution of the stream), which can significantly affect the ability to force air into a building^7^. Additionally, it can be noted that many fans of a commercial design can adjust the inclination of the impeller axis in relation to the ground. There is a noticeable lack of tests of the position of the fans in a distance greater than 1–3 m, along with the change of the impeller position relative to the ground for studies of smoke ventilation of buildings. There are also no air flow analyses at various points of the door openings, which may change depending on the shape of the stream generated by the fan (using 50 measuring points). Regardless of the quality of the used mobile positive pressure fans, the ability to properly operate these devices is of great importance for the effectiveness of the rescue operation. The correct positioning of the device may significantly affect the possibility of carrying out a rescue operation, affecting the duration of this action, and ultimately the energy consumption of the device, which in extreme situations may shorten the time of using the device in a rescue operation.

The article aims to determine the effect of the settings of four commercial and popularly used mobile positive pressure fans (with different drive power characteristics) on air flow in a door opening. The article analyzes the influence of setting two parameters, the distance between the fan and the inlet opening, and the influence of the impeller inclination angle to the ground plane. Air flow was tested at fifty points in a door-sized opening. Specialized research equipment was used for this. It was also checked as to whether these fans are characterized by an individual acceptance of settings, or whether universal guidelines for the settings of these devices can be proposed. It was also determined how the fan settings affect the energy consumption of the building ventilation process. The tests can provide new information on the use of the fans and provide test results for the validation of simulation models. The novelty of the manuscript is a study describing the impact of the positioning of mobile emergency ventilators on the efficiency and effectiveness of their work.

## Material and methods

**Figure 1:**
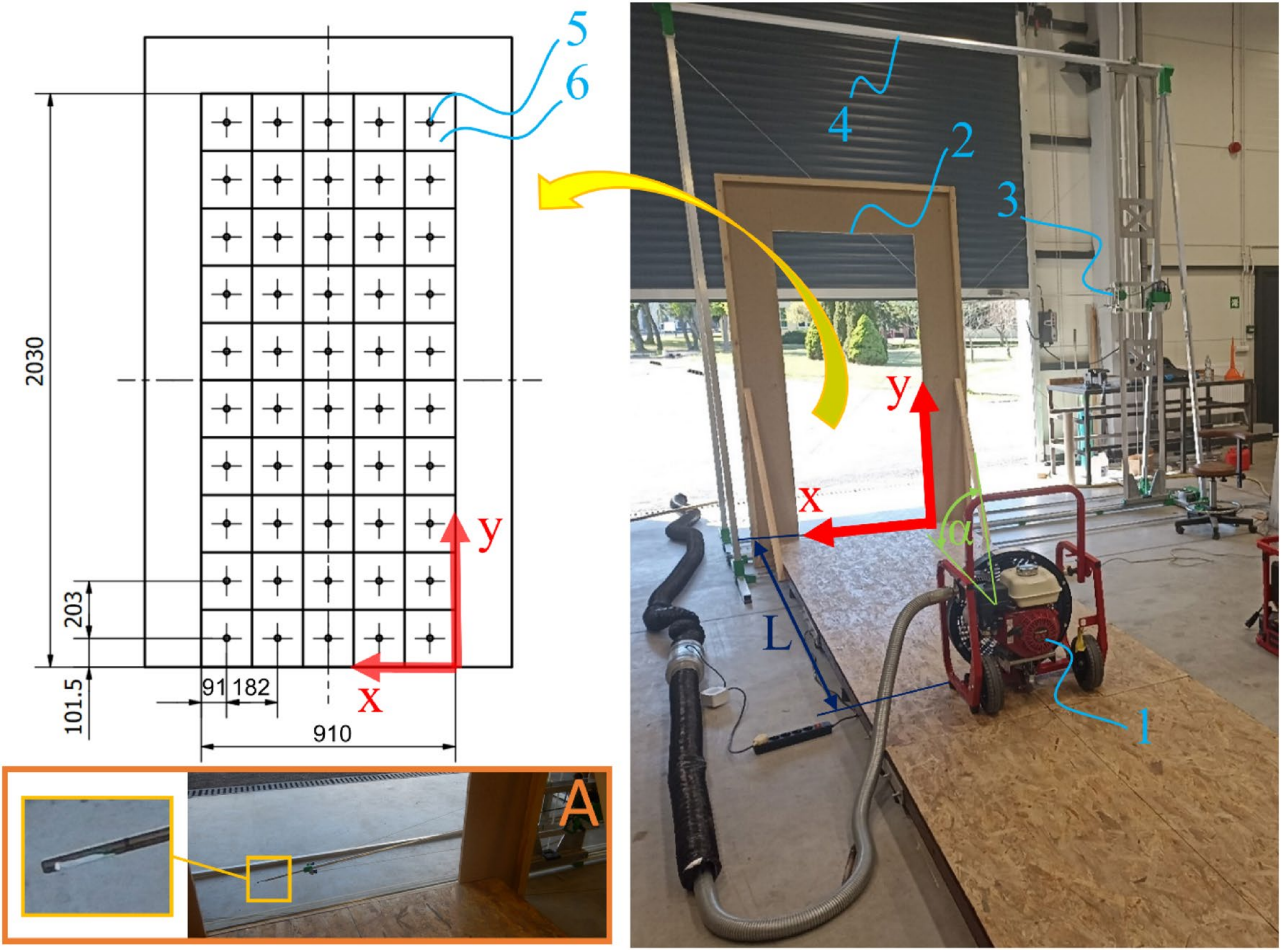
Test stand, where 1—positive pressure ventilator, 2—door opening, 3—measuring module of the test stand, 4—test stand frame and guides for transporting the measuring module, 5—measuring point, 6—measurement area of the door opening for which the test is performed at the measurement point, A—measuring probe TSI hot-wire anemometer moving on the surface of the measuring plane (the gate in the research hall was open during the research).

The tests of the air flow velocity profile on the surface of the door opening were carried out on a dedicated stand for assessing the characteristics of the air stream velocity profiles generated by mobile positive pressure ventilators in an open flow. The stand is the equipment of the Scientific and Research Center for Fire Protection—National Research Institute, Jozefów, Poland. An important aspect of the stand’s operation is the fact that during the tests it allows for taking into account the geometric parameters related to fan positioning, i.e., setting the distance and impeller inclination angle. The measuring plane (enabling the probing of the surface of 2880 × 3070 mm) was combined with an obstacle imitating a door opening with dimensions of 2.03 × 0.91 m^15^, and 50 measurement points were located on its surface. The points were distributed evenly over the entire surface of the opening—based on ISO 5221^16^ (a method of even surface traversing). The distribution of the measurement points in the door opening and the reference point consistent with the zero point of the adopted coordinate system are shown in Fig. 1. The measuring module is equipped with a TSI type 8455 thermo-resistive anemometer with a measuring range of 0.127—50 m/s and accuracy of approx. 1% of the reading. The length of the TSI anemometer probe was 1200 mm. Stable mounting of the anemometer to the movable transport element allowed automatic and repeatable changes of the measurement points. The probe was transported with stepper motors (with positioning accuracy not less than 0.1 mm).

**Figure 2:**
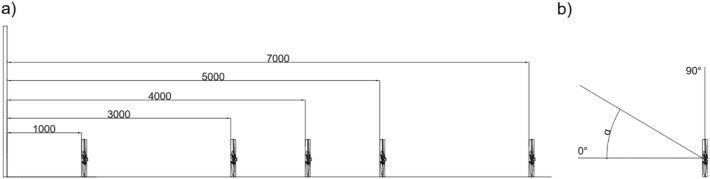
Diagram of the research program, where: (**a**) distance of the positive pressure ventilator from the door opening, (**b**) the angle of inclination of the fan impeller relative to the ground, where α = 0°—the position of the fan impeller is parallel to the ground.

The research program was configured as follows: the acquisition frequency was 10 Hz and the duration of the measurement of one point was 300 s. During the tests, for the fan which was arranged in front of the test stand, the velocity profile was assessed. The surface of the fan rotor was directed to the measurement plane of the door opening. The velocity profile measurement was performed for variables distances, i.e. 1 m, 3 m, 4 m, 5 m and 7 m (Fig. 2a) and the rotor inclination angle in the range from 0° to 18° (Fig. 2b). The rotor inclination angles were set in accordance with the positions recommended by the manufacturers—the fan constructions have four-section position adjustment mechanisms. The values of the angles for the tested structures are presented in Table 1. During the tests, the volumetric air flow *Q*, blowing onto the surface of the door opening, was also estimated from the relationship of the average value of the air stream velocity *V* and the door opening surface *S*, in accordance with Eq. (1).

1 $$Q = V \cdot S, \left[ {\frac{{m^{3} }}{h} = \frac{m}{h} \cdot m^{2} } \right]$$

where:

**Table 1:** Technical parameters of the tested positive pressure ventilators.

Parameter	1	2	3	4
Model	EX 50Li	FOGO MW 22	GX 350	GX 500
Manufacturer (city, country)	Ramfan (Spring Valley, USA)	FOGO Sp. z o.o. (Wilkowice, Poland)	Ramfan (Spring Valley, USA)	Ramfan (Spring Valley, USA)
Type of drive unit	Electric engine—with battery	Combustion engine with spark ignition	Combustion engine with spark ignition	Combustion engine with spark ignition
Flow straightener on the fan impeller	+	−	+	+
Power of the drive unit	0.6 kW	4.4 kW	4.1 kW	6.3 kW
Rotational speed when working at full power	2790 rpm	3289 rpm	3585 rpm	3230 rpm
Impeller diameter	460 mm	495 mm	460 mm	600 mm
Quantity of the rotor blades	5	8	9	7
adjustment range of the impeller angles (recommended positions by manufacturer)	0°–18° (0°, 6°, 12°, 18°)	0°–16° (0°, 6°, 11°, 16°)	0°–18° (0°, 6°, 12°, 18°)	0°–17° (0°, 5°, 11°, 17°)

**Table 2:** Air flow velocity in the door opening for fan 1, where: x—position of the measuring point in relation to the x-axis and, y—position of the measuring point in relation to the y-axis.

Distance of the positive pressure ventilator 1 from the door opening 1m The inclination angle of the positive pressure ventilator impeller relative to the ground 0°											
		x (mm)									
		91		273		455		637		819	
		AVG	SD	AVG	SD	AVG	SD	AVG	SD	AVG	SD
y (mm)	101.5	0.534	0.11	3.214	0.22	10.788	0.27	4.332	0.37	0.827	0.07
	304.5	0.785	0.14	9.667	0.14	12.821	0.10	10.436	0.51	0.957	0.29
	507.5	0.424	0.04	1.849	0.24	6.198	0.31	2.240	0.36	0.346	0.03
	710.5	0.364	0.11	0.350	0.07	0.308	0.02	0.433	0.11	0.330	0.10
	913.5	0.236	0.05	0.232	0.03	0.258	0.04	0.282	0.04	0.283	0.14
	1116.5	0.183	0.01	0.216	0.04	0.231	0.02	0.294	0.06	0.208	0.02
	1319.5	0.195	0.03	0.137	0.02	0.257	0.03	0.278	0.05	0.198	0.01
	1522.5	0.130	0.02	0.154	0.02	0.203	0.02	0.226	0.06	0.187	0.02
	1725.5	0.110	0.00	0.163	0.05	0.241	0.05	0.189	0.04	0.163	0.02
	1928.5	0.203	0.02	0.195	0.01	0.205	0.03	0.217	0.03	0.160	0.04

Distance of the positive pressure ventilator 1 from the door opening 1m The inclination angle of the positive pressure ventilator impeller relative to the ground 6°											
		x (mm)									
		91		273		455		637		819	
		AVG	SD	AVG	SD	AVG	SD	AVG	SD	AVG	SD
y (mm)	101.5	0.646	0.11	1.357	0.26	3.739	0.23	0.8	0.12	0.927	0.19
	304.5	0.652	0.09	7.434	0.42	14.376	0.15	6.898	0.57	0.683	0.11
	507.5	0.532	0.05	7.439	0.71	15.005	0.12	7.916	0.37	1.039	0.18
	710.5	0.389	0.08	0.74	0.11	1.462	0.21	1.24	0.28	0.502	0.12
	913.5	0.389	0.19	0.307	0.06	0.754	0.23	0.536	0.30	0.331	0.09
	1116.5	0.305	0.10	0.312	0.10	0.232	0.03	0.282	0.10	0.269	0.04
	1319.5	0.239	0.02	0.327	0.14	0.201	0.02	0.228	0.06	0.182	0.03
	1522.5	0.219	0.03	0.219	0.02	0.239	0.06	0.145	0.03	0.133	0.02
	1725.5	0.205	0.03	0.206	0.04	0.256	0.07	0.203	0.02	0.198	0.02
	1928.5	0.192	0.01	0.234	0.04	0.299	0.06	0.229	0.02	0.174	0.04

Distance of the positive pressure ventilator 1 from the door opening 1m The inclination angle of the positive pressure ventilator impeller relative to the ground 12°											
		x (mm)									
		91		273		455		637		819	
		AVG	SD	AVG	SD	AVG	SD	AVG	SD	AVG	SD
y (mm)	101.5	0.96	0.35	1.447	0.31	0.949	0.24	0.807	0.32	1.058	0.16
	304.5	0.499	0.09	3.384	0.57	8.149	0.54	3.599	0.57	0.56	0.07
	507.5	1.195	0.26	9.505	0.47	13.184	0.17	10.252	0.50	0.645	0.10
	710.5	0.391	0.11	5.118	0.34	11.231	0.20	3.817	0.62	0.626	0.14
	913.5	0.391	0.07	0.486	0.12	0.633	0.18	0.45	0.11	0.384	0.05
	1116.5	0.256	0.05	0.365	0.07	0.449	0.08	0.361	0.06	0.41	0.11
	1319.5	0.321	0.11	0.349	0.14	0.323	0.07	0.336	0.11	0.436	0.08
	1522.5	0.227	0.03	0.302	0.04	0.306	0.03	0.393	0.10	0.311	0.08
	1725.5	0.209	0.02	0.342	0.08	0.165	0.03	0.206	0.04	0.299	0.06
	1928.5	0.21	0.02	0.229	0.03	0.174	0.02	0.161	0.02	0.21	0.04

Distance of the positive pressure ventilator 1 from the door opening 1m The inclination angle of the positive pressure ventilator impeller relative to the ground 18°											
		x (mm)									
		91		273		455		637		819	
		AVG	SD	AVG	SD	AVG	SD	AVG	SD	AVG	SD
y (mm)	101.5	1.424	0.34	0.521	0.23	0.615	0.19	0.964	0.27	1.102	0.13
	304.5	0.961	0.31	1.367	0.31	1.315	0.21	0.509	0.13	1.06	0.14
	507.5	0.743	0.11	5.728	0.54	13.928	0.34	6.023	0.33	0.558	0.08
	710.5	0.737	0.18	9.441	0.86	14.618	0.23	9.035	0.46	0.572	0.08
	913.5	0.519	0.09	2.619	0.33	5.611	0.77	2.432	0.32	0.552	0.15
	1116.5	0.266	0.03	0.313	0.11	0.353	0.03	0.274	0.01	0.27	0.05
	1319.5	0.33	0.06	0.782	0.14	0.532	0.22	0.223	0.04	0.342	0.06
	1522.5	0.232	0.05	0.223	0.03	0.498	0.16	0.786	0.21	0.261	0.04
	1725.5	0.166	0.02	0.338	0.10	0.334	0.14	0.334	0.08	0.256	0.05
	1928.5	0.219	0.05	0.202	0.02	0.235	0.03	0.252	0.05	0.301	0.07

Distance of the positive pressure ventilator 1 from the door opening 3 m The inclination angle of the positive pressure ventilator impeller relative to the ground 0°											
		x (mm)									
		91		273		455		637		819	
		AVG	SD	AVG	SD	AVG	SD	AVG	SD	AVG	SD
y (mm)	101.5	4.608	0.44	7.603	0.34	9.112	0.34	6.591	0.37	3.874	0.48
	304.5	4.671	0.50	8.444	0.37	9.769	0.31	7.163	0.47	3.881	0.55
	507.5	3.847	0.43	5.523	0.42	6.903	0.42	4.48	0.67	2.374	0.58
	710.5	0.793	0.46	3.039	0.66	2.485	0.52	1.572	0.47	0.905	0.24
	913.5	0.793	0.19	0.696	0.14	0.411	0.09	0.479	0.11	0.393	0.06
	1116.5	0.506	0.20	0.289	0.05	1.021	0.33	0.404	0.11	0.398	0.13
	1319.5	0.679	0.31	0.714	0.30	0.485	0.11	0.371	0.16	0.524	0.19
	1522.5	0.344	0.10	0.313	0.04	0.856	0.21	0.649	0.35	0.767	0.27
	1725.5	0.729	0.53	0.283	0.06	0.261	0.04	0.296	0.06	0.366	0.14
	1928.5	0.295	0.07	0.319	0.04	0.388	0.11	0.838	0.27	0.271	0.05

Distance of the positive pressure ventilator 1 from the door opening 3 m The inclination angle of the positive pressure ventilator impeller relative to the ground 6°											
		x (mm)									
		91		273		455		637		819	
		AVG	SD	AVG	SD	AVG	SD	AVG	SD	AVG	SD
y (mm)	101.5	1.971	0.94	3.063	0.43	3.688	0.67	1.933	0.46	0.973	0.30
	304.5	2.346	0.57	5.22	0.73	6.092	0.31	4.253	0.51	2.027	0.37
	507.5	3.521	0.54	7.233	0.42	9.182	0.30	6.581	0.43	2.907	0.70
	710.5	3.467	0.42	7.233	0.48	9.147	0.38	6.245	0.49	2.414	0.66
	913.5	3.467	0.53	5.282	0.68	5.734	0.52	4.046	0.67	1.615	0.57
	1116.5	1.335	0.56	1.794	0.70	2.026	0.67	1.41	0.42	0.871	0.26
	1319.5	0.484	0.09	1.158	0.19	0.605	0.19	0.569	0.15	1.063	0.36
	1522.5	0.813	0.25	0.931	0.27	0.98	0.39	0.959	0.37	0.977	0.37
	1725.5	0.805	0.25	0.515	0.20	0.803	0.48	0.75	0.39	0.801	0.14
	1928.5	0.561	0.19	0.746	0.31	0.678	0.20	0.511	0.14	0.371	0.08

Distance of the positive pressure ventilator 1 from the door opening 3 m The inclination angle of the positive pressure ventilator impeller relative to the ground 12°											
		x (mm)									
		91		273		455		637		819	
		AVG	SD	AVG	SD	AVG	SD	AVG	SD	AVG	SD
y (mm)	101.5	1.122	0.36	1.14	0.49	1.529	0.25	1.135	0.38	0.868	0.34
	304.5	1.15	0.33	1.06	0.15	1.334	0.31	1.292	0.29	0.663	0.13
	507.5	2.301	0.33	3.089	0.48	2.928	0.43	2.737	0.82	1.014	0.18
	710.5	4.519	0.54	6.833	0.36	7.463	0.52	6.176	0.60	1.81	0.42
	913.5	4.519	0.42	8.088	0.44	9.569	0.14	6.747	0.38	3.075	0.43
	1116.5	4.235	0.39	6.83	0.58	7.311	0.54	5.07	0.74	2.806	0.57
	1319.5	2.782	0.30	4.201	0.57	4.448	0.53	2.832	0.63	1.022	0.34
	1522.5	1.038	0.31	1.375	0.41	0.873	0.28	0.692	0.18	0.551	0.10
	1725.5	0.553	0.14	0.395	0.09	0.332	0.08	0.301	0.05	0.356	0.05
	1928.5	0.297	0.06	0.237	0.02	0.341	0.11	0.264	0.04	0.343	0.16

Distance of the positive pressure ventilator 1 from the door opening 3 m The inclination angle of the positive pressure ventilator impeller relative to the ground 18°											
		x (mm)									
		91		273		455		637		819	
		AVG	SD	AVG	SD	AVG	SD	AVG	SD	AVG	SD
y (mm)	101.5	0.6	0.13	1.104	0.18	0.886	0.36	1.381	0.06	0.486	0.16
	304.5	1.15	0.25	1.061	0.39	1.092	0.28	0.361	0.18	0.367	0.11
	507.5	0.56	0.13	0.737	0.15	0.636	0.14	0.432	0.09	0.312	0.07
	710.5	2.81	0.22	1.319	0.32	1.404	0.33	1.451	0.50	0.764	0.19
	913.5	2.81	0.74	3.899	0.76	4.387	0.23	3.493	0.34	1.695	0.22
	1116.5	3.741	0.51	6.362	0.34	7.567	0.41	6.047	0.81	3.024	0.37
	1319.5	3.47	0.72	7.817	0.33	9.541	0.52	7.345	0.63	3.199	0.41
	1522.5	4.361	0.37	6.419	0.56	7.518	0.22	5.231	0.77	2.535	0.36
	1725.5	2.004	0.25	3.955	0.45	3.748	0.72	3.226	0.71	2.118	0.34
	1928.5	0.696	0.24	1.104	0.26	0.931	0.48	0.8	0.26	0.669	0.22

Distance of the positive pressure ventilator 1 from the door opening 4 m The inclination angle of the positive pressure ventilator impeller relative to the ground 0°											
		x (mm)									
		91		273		455		637		819	
		AVG	SD	AVG	SD	AVG	SD	AVG	SD	AVG	SD
y (mm)	101.5	4.732	0.42	7.375	0.35	7.856	0.32	6.45	0.35	4.7	0.30
	304.5	5.078	0.57	7.156	0.27	7.672	0.37	6.051	0.24	3.178	0.43
	507.5	4.239	0.41	5.949	0.38	6.078	0.40	4.503	0.74	3.007	0.41
	710.5	1.366	0.40	2.802	0.75	3.212	0.56	3.418	0.54	2.37	0.42
	913.5	1.366	0.41	1.09	0.19	1.089	0.24	0.998	0.34	0.91	0.15
	1116.5	0.626	0.10	0.641	0.28	0.926	0.29	0.786	0.18	0.597	0.15
	1319.5	0.429	0.07	0.598	0.17	0.738	0.14	0.758	0.31	1.011	0.27
	1522.5	0.658	0.20	1.116	0.34	0.61	0.21	0.316	0.07	0.682	0.15
	1725.5	0.32	0.06	0.524	0.13	0.312	0.06	1.175	0.35	0.434	0.19
	1928.5	0.257	0.04	0.551	0.20	0.261	0.04	0.465	0.21	0.266	0.05

Distance of the positive pressure ventilator 1 from the door opening 4 m The inclination angle of the positive pressure ventilator impeller relative to the ground 6°											
		x (mm)									
		91		273		455		637		819	
		AVG	SD	AVG	SD	AVG	SD	AVG	SD	AVG	SD
y (mm)	101.5	2.048	0.40	3.051	0.57	2.89	0.41	2.545	0.59	1.574	0.52
	304.5	3.269	0.83	4.66	0.53	3.97	0.31	3.306	0.36	1.707	0.57
	507.5	4.181	0.45	6.114	0.46	6.51	0.62	4.45	0.43	3.067	0.51
	710.5	4.798	0.66	7.225	0.28	7.271	0.35	5.334	0.59	3.042	0.50
	913.5	4.798	0.52	6.433	0.37	6.657	0.30	4.329	0.35	2.062	0.51
	1116.5	2.675	0.71	3.287	0.66	3.839	0.65	3.072	0.71	1.583	0.48
	1319.5	1.864	0.52	1.706	0.26	1.391	0.57	1.051	0.31	0.907	0.41
	1522.5	1.074	0.17	0.59	0.09	0.611	0.20	0.439	0.10	0.373	0.06
	1725.5	0.521	0.09	0.497	0.11	0.282	0.04	0.453	0.27	0.715	0.21
	1928.5	0.225	0.03	0.25	0.03	0.284	0.06	0.684	0.20	0.481	0.14

Distance of the positive pressure ventilator 1 from the door opening 4 m The inclination angle of the positive pressure ventilator impeller relative to the ground 12°											
		x (mm)									
		91		273		455		637		819	
		AVG	SD	AVG	SD	AVG	SD	AVG	SD	AVG	SD
y (mm)	101.5	0.572	0.10	0.69	0.18	1.422	0.29	1.467	0.16	1.11	0.16
	304.5	0.86	0.24	0.746	0.13	1.001	0.25	0.688	0.09	0.897	0.28
	507.5	1.195	0.53	1.194	0.37	1.486	0.33	1.818	0.73	0.995	0.21
	710.5	4.526	0.69	4.144	0.47	3.837	0.60	3.155	0.48	1.923	0.69
	913.5	4.526	0.62	5.974	0.39	5.82	0.38	4.655	0.53	2.61	0.66
	1116.5	4.445	0.47	6.999	0.46	7.473	0.37	6.21	0.33	3.528	0.63
	1319.5	4.423	0.59	5.866	0.62	6.26	0.60	4.363	1.03	3.232	0.47
	1522.5	2.75	0.51	4.053	0.37	4.542	0.32	2.529	0.51	2.072	0.62
	1725.5	0.975	0.50	1.81	0.26	1.547	0.57	1.764	0.72	1.419	0.47
	1928.5	1.059	0.19	0.954	0.43	1.174	0.31	0.334	0.05	0.394	0.13

Distance of the positive pressure ventilator 1 from the door opening 4 m The inclination angle of the positive pressure ventilator impeller relative to the ground 18°											
		x (mm)									
		91		273		455		637		819	
		AVG	SD	AVG	SD	AVG	SD	AVG	SD	AVG	SD
y (mm)	101.5	0.539	0.15	1.001	0.13	0.845	0.51	1.178	0.36	0.799	0.24
	304.5	0.668	0.09	0.865	0.13	0.765	0.10	0.848	0.16	0.633	0.12
	507.5	0.6	0.21	0.648	0.17	0.282	0.04	0.413	0.11	0.445	0.14
	710.5	0.889	0.16	0.728	0.25	0.621	0.18	0.574	0.17	0.418	0.07
	913.5	0.889	0.18	0.627	0.16	1.389	0.33	0.937	0.39	0.591	0.20
	1116.5	2.294	0.35	2.692	0.48	2.711	0.66	2.828	0.46	1.926	0.42
	1319.5	4.346	0.48	5.017	0.30	5.359	1.03	4.434	0.70	2.418	0.56
	1522.5	4.32	0.41	6.342	0.32	6.445	0.37	5.514	0.57	3.886	0.59
	1725.5	4.831	0.40	5.962	0.30	6.994	0.53	6.153	0.52	3.586	0.29
	1928.5	3.759	0.37	5.404	0.31	5.676	0.44	4.762	0.48	3.277	0.26

Distance of the positive pressure ventilator 1 from the door opening 5 m The inclination angle of the positive pressure ventilator impeller relative to the ground 0°											
		x (mm)									
		91		273		455		637		819	
		AVG	SD	AVG	SD	AVG	SD	AVG	SD	AVG	SD
y (mm)	101.5	5.615	0.30	6.232	0.30	6.574	0.26	6.121	0.39	4.899	0.38
	304.5	5.558	0.33	6.307	0.45	6.723	0.31	5.751	0.26	3.858	0.67
	507.5	4.312	0.45	4.975	0.51	5.426	0.48	4.555	0.41	3.06	0.49
	710.5	2.57	0.47	3.802	0.46	4.03	0.46	3.028	0.59	2.246	0.43
	913.5	2.57	0.47	1.866	0.46	1.807	0.43	1.56	0.46	1.151	0.33
	1116.5	1.11	0.30	0.844	0.13	0.962	0.25	0.932	0.21	1.024	0.22
	1319.5	0.592	0.20	0.642	0.21	0.742	0.12	0.658	0.19	0.943	0.28
	1522.5	0.408	0.04	0.78	0.20	1.12	0.23	0.386	0.05	1.177	0.24
	1725.5	0.733	0.36	0.745	0.27	0.536	0.19	0.485	0.11	0.98	0.27
	1928.5	0.426	0.12	0.582	0.20	0.281	0.05	0.681	0.20	0.352	0.13

Distance of the positive pressure ventilator 1 from the door opening 5 m The inclination angle of the positive pressure ventilator impeller relative to the ground 6°											
		x (mm)									
		91		273		455		637		819	
		AVG	SD	AVG	SD	AVG	SD	AVG	SD	AVG	SD
y (mm)	101.5	2.18	0.54	3.047	0.51	2.673	0.50	2.663	0.47	2.104	0.50
	304.5	3.348	0.62	3.962	0.53	3.715	0.61	2.879	0.57	1.846	0.22
	507.5	3.851	0.40	5.117	0.41	5.253	0.17	4.461	0.78	3.384	0.41
	710.5	4.715	0.57	5.585	0.53	5.723	0.47	4.163	0.51	2.911	0.55
	913.5	4.715	0.34	5.391	0.34	5.637	0.21	4.668	0.37	3.487	0.77
	1116.5	3.943	0.49	4.592	0.49	3.763	0.52	3.157	0.62	2.72	0.61
	1319.5	3.365	0.35	2.682	0.25	2.56	0.48	1.957	0.34	1.107	0.41
	1522.5	0.993	0.23	1.359	0.34	1.218	0.27	0.646	0.09	0.809	0.28
	1725.5	0.676	0.19	0.495	0.10	0.672	0.17	0.675	0.17	0.775	0.16
	1928.5	0.438	0.10	0.718	0.10	0.365	0.07	0.44	0.10	0.378	0.10

Distance of the positive pressure ventilator 1 from the door opening 5 m The inclination angle of the positive pressure ventilator impeller relative to the ground 12°											
		x (mm)									
		91		273		455		637		819	
		AVG	SD	AVG	SD	AVG	SD	AVG	SD	AVG	SD
y (mm)	101.5	0.696	0.25	0.681	0.21	1	0.31	0.991	0.21	0.838	0.22
	304.5	0.905	0.22	0.807	0.41	0.862	0.18	0.688	0.28	0.657	0.21
	507.5	1.81	0.34	1.853	0.54	1.405	0.32	1.273	0.37	1.497	0.70
	710.5	3.283	0.28	2.74	0.44	2.186	0.54	1.853	0.48	1.903	0.37
	913.5	3.283	0.50	3.789	0.43	3.881	0.25	3.321	0.39	2.209	0.53
	1116.5	4.123	0.35	5.267	0.29	5.214	0.32	4.473	0.46	2.885	0.41
	1319.5	4.42	0.42	5.712	0.30	5.786	0.20	4.834	0.45	3.079	0.77
	1522.5	4.551	0.43	5.068	0.29	5.255	0.58	3.941	0.37	3.192	0.26
	1725.5	3.452	0.48	4.185	0.26	2.727	0.39	3.195	0.39	2.541	0.35
	1928.5	2.643	0.57	1.699	0.63	2.44	0.18	2.483	0.49	1.92	0.27

Distance of the positive pressure ventilator 1 from the door opening 5 m The inclination angle of the positive pressure ventilator impeller relative to the ground 18°											
		x (mm)									
		91		273		455		637		819	
		AVG	SD	AVG	SD	AVG	SD	AVG	SD	AVG	SD
y (mm)	101.5	0.702	0.14	1.067	0.50	0.607	0.23	0.879	0.12	0.556	0.20
	304.5	0.351	0.09	0.748	0.09	0.822	0.22	0.233	0.03	0.301	0.05
	507.5	0.385	0.06	0.295	0.07	1.183	0.14	0.937	0.34	1.01	0.28
	710.5	0.577	0.12	0.42	0.09	0.617	0.10	0.737	0.23	0.627	0.20
	913.5	0.577	0.23	1.129	0.33	0.734	0.33	1.058	0.27	0.885	0.33
	1116.5	1.538	0.39	1.331	0.43	2.169	0.48	1.666	0.36	1.881	0.45
	1319.5	2.663	0.32	3.417	0.69	3.729	0.67	2.838	0.59	1.469	0.37
	1522.5	3.732	0.17	3.665	0.39	5.373	0.39	3.241	0.47	2.945	0.77
	1725.5	4.564	0.31	5.113	0.28	5.033	0.37	4.443	0.27	4.026	0.36
	1928.5	4.933	0.39	5.744	0.24	5.899	0.27	5.428	0.25	4.402	0.36

Distance of the positive pressure ventilator 1 from the door opening 7 m The inclination angle of the positive pressure ventilator impeller relative to the ground 0°											
		x (mm)									
		91		273		455		637		819	
		AVG	SD	AVG	SD	AVG	SD	AVG	SD	AVG	SD
y (mm)	101.5	4.89	0.26	5.557	0.34	5.608	0.24	4.535	0.39	3.804	0.47
	304.5	4.685	0.27	5.272	0.33	4.962	0.30	4.307	0.28	3.249	0.54
	507.5	4.382	0.33	4.327	0.43	4.388	0.28	4.122	0.57	2.807	0.54
	710.5	2.777	0.52	3.337	0.46	3.416	0.33	3.198	0.53	2.061	0.39
	913.5	2.777	0.29	2.521	0.47	2.044	0.58	2.01	0.50	1.618	0.29
	1116.5	2.224	0.37	1.325	0.30	1.661	0.51	1.439	0.56	0.789	0.28
	1319.5	1.454	0.38	1.253	0.30	1.169	0.36	0.935	0.20	0.646	0.15
	1522.5	0.542	0.15	0.72	0.14	0.82	0.10	0.637	0.16	0.835	0.34
	1725.5	0.677	0.14	0.579	0.08	0.759	0.27	0.603	0.17	0.841	0.31
	1928.5	0.363	0.06	0.54	0.18	0.662	0.17	0.498	0.11	0.605	0.10

Distance of the positive pressure ventilator 1 from the door opening 7 m The inclination angle of the positive pressure ventilator impeller relative to the ground 6°											
		x (mm)									
		91		273		455		637		819	
		AVG	SD	AVG	SD	AVG	SD	AVG	SD	AVG	SD
y (mm)	101.5	3.085	0.45	2.827	0.46	2.544	0.40	2.508	0.55	1.874	0.30
	304.5	2.795	0.53	3.618	0.35	2.977	0.51	2.233	0.33	2.441	0.60
	507.5	3.294	0.57	3.919	0.19	3.473	0.38	2.81	0.71	2.819	0.54
	710.5	3.47	0.26	3.776	0.16	3.747	0.43	3.009	0.54	2.449	0.41
	913.5	3.47	0.38	4.15	0.29	3.935	0.39	3.839	0.44	2.733	0.35
	1116.5	2.967	0.53	3.354	0.31	3.651	0.34	3.625	0.29	2.609	0.28
	1319.5	3.308	0.52	3.425	0.40	3.308	0.34	2.641	0.47	2.09	0.56
	1522.5	2.342	0.66	2.658	0.48	2.762	0.40	2.043	0.47	1.471	0.25
	1725.5	2.301	0.53	1.967	0.41	1.654	0.36	1.022	0.24	1.359	0.59
	1928.5	1.65	0.53	1.239	0.26	1.241	0.41	0.845	0.16	0.562	0.23

Distance of the positive pressure ventilator 1 from the door opening 7 m The inclination angle of the positive pressure ventilator impeller relative to the ground 12°											
		x (mm)									
		91		273		455		637		819	
		AVG	SD	AVG	SD	AVG	SD	AVG	SD	AVG	SD
y (mm)	101.5	0.85	0.18	0.799	0.26	0.935	0.36	0.703	0.29	0.662	0.21
	304.5	0.633	0.16	0.653	0.30	0.599	0.14	0.476	0.11	0.58	0.20
	507.5	0.944	0.35	0.897	0.18	0.77	0.32	0.881	0.31	0.807	0.31
	710.5	2.367	0.42	1.452	0.45	1.196	0.28	1.359	0.36	1.124	0.29
	913.5	2.367	0.41	2.376	0.50	1.9	0.25	1.941	0.56	1.825	0.35
	1116.5	2.618	0.20	2.384	0.27	2.886	0.47	2.977	0.63	2.038	0.38
	1319.5	3.317	0.36	3.663	0.34	3.456	0.49	2.822	0.48	2.477	0.39
	1522.5	3.66	0.31	3.834	0.32	3.961	0.23	3.546	0.51	2.207	0.40
	1725.5	3.37	0.30	3.977	0.27	4.217	0.24	3.724	0.25	3.106	0.33
	1928.5	3.929	0.38	3.915	0.29	3.395	0.29	3.45	0.37	2.703	0.33

Distance of the positive pressure ventilator 1 from the door opening 7 m The inclination angle of the positive pressure ventilator impeller relative to the ground 18°											
		x (mm)									
		91		273		455		637		819	
		AVG	SD	AVG	SD	AVG	SD	AVG	SD	AVG	SD
y (mm)	101.5	0.658	0.14	0.689	0.15	0.948	0.22	1.007	0.17	0.764	0.13
	304.5	0.557	0.09	0.637	0.19	1.053	0.15	0.619	0.27	0.704	0.13
	507.5	0.389	0.06	0.483	0.11	0.495	0.18	0.585	0.15	0.419	0.07
	710.5	0.477	0.20	0.395	0.08	0.302	0.04	0.347	0.06	0.43	0.11
	913.5	0.477	0.11	0.596	0.17	0.302	0.04	0.29	0.08	0.318	0.04
	1116.5	0.797	0.25	0.59	0.12	0.705	0.21	0.377	0.15	0.546	0.19
	1319.5	1.216	0.32	0.722	0.23	1.087	0.19	0.94	0.21	0.738	0.18
	1522.5	1.487	0.37	1.689	0.24	1.834	0.27	1.258	0.24	1.206	0.27
	1725.5	2.124	0.35	2.088	0.45	2.232	0.30	2.295	0.49	1.894	0.45
	1928.5	1.715	0.44	1.25	0.31	1.296	0.37	1.163	0.29	1.792	0.41

AVG—arithmetic mean of the flow rate, SD—standard deviation taken as the measurement error.

**Table 3:** Air flow velocity in the door opening for fan 2, where x—position of the measuring point in relation to the x-axis and, y—position of the measuring point in relation to the y-axis.

Distance of the positive pressure ventilator 2 from the door opening 1m The inclination angle of the positive pressure ventilator impeller relative to the ground 0°											
		x (mm)									
		91		273		455		637		819	
		AVG	SD	AVG	SD	AVG	SD	AVG	SD	AVG	SD
y (mm)	101.5	6.719	0.82	14.552	0.95	9.015	0.89	13.083	0.42	9.666	0.53
	304.5	7.299	1.24	14.031	1.08	5.895	0.75	17.249	0.70	10.355	1.49
	507.5	11.189	1.25	9.521	1.38	17.269	0.78	15.549	1.78	7.628	1.29
	710.5	0.436	0.52	1.753	0.48	6.122	2.20	1.843	0.42	0.799	0.15
	913.5	0.436	0.18	0.312	0.03	0.584	0.21	0.77	0.45	0.593	0.22
	1116.5	0.38	0.15	0.345	0.07	0.407	0.21	0.779	0.28	0.883	0.44
	1319.5	0.286	0.04	0.429	0.18	0.285	0.07	0.264	0.03	0.291	0.04
	1522.5	0.945	0.25	0.761	0.29	0.686	0.24	0.544	0.16	0.437	0.10
	1725.5	0.529	0.22	0.277	0.08	0.341	0.14	0.495	0.13	0.292	0.14
	1928.5	0.244	0.04	0.312	0.08	0.288	0.10	0.225	0.05	0.204	0.03

Distance of the positive pressure ventilator 2 from the door opening 1m The inclination angle of the positive pressure ventilator impeller relative to the ground 6°											
		x (mm)									
		91		273		455		637		819	
		AVG	SD	AVG	SD	AVG	SD	AVG	SD	AVG	SD
y (mm)	101.5	3.765	0.59	9.089	0.97	5.584	0.62	8.183	0.67	7.185	0.82
	304.5	7.867	2.28	15.134	1.36	10.295	0.97	12.39	0.63	9.035	1.28
	507.5	12.372	1.13	15.044	1.89	8.323	0.93	16.077	0.94	14.238	0.55
	710.5	0.622	1.35	4.552	0.84	13.96	1.94	6.278	1.40	1.65	0.25
	913.5	0.622	0.24	0.839	0.17	1.498	0.56	0.957	0.30	0.849	0.16
	1116.5	0.805	0.32	0.945	0.27	1.181	0.30	0.598	0.19	0.801	0.32
	1319.5	0.891	0.28	0.616	0.28	0.559	0.31	0.965	0.38	0.67	0.22
	1522.5	0.846	0.19	0.567	0.17	1.52	0.35	0.71	0.17	0.953	0.43
	1725.5	0.875	0.30	0.554	0.17	0.637	0.24	0.73	0.19	0.751	0.24
	1928.5	0.334	0.10	0.658	0.11	0.875	0.18	0.639	0.18	0.439	0.13

Distance of the positive pressure ventilator 2 from the door opening 1m The inclination angle of the positive pressure ventilator impeller relative to the ground 11°											
		x (mm)									
		91		273		455		637		819	
		AVG	SD	AVG	SD	AVG	SD	AVG	SD	AVG	SD
y (mm)	101.5	2.705	0.65	3.884	1.40	4.898	0.64	4.348	0.99	5.516	0.82
	304.5	4.583	0.99	12.506	1.34	9.203	1.63	8.426	0.63	6.808	1.11
	507.5	7.22	1.10	14.504	1.73	6.101	0.76	12.606	0.68	10.627	1.22
	710.5	1.674	0.71	11.702	2.63	14.089	1.33	15.051	1.12	8.357	1.43
	913.5	1.674	0.51	5.066	1.94	8.549	1.08	4.422	1.85	2.124	0.90
	1116.5	0.502	0.12	0.599	0.15	0.971	0.31	1.284	0.53	0.564	0.11
	1319.5	0.512	0.17	0.413	0.11	0.416	0.06	0.47	0.11	0.355	0.06
	1522.5	0.483	0.10	0.259	0.04	0.583	0.19	0.568	0.16	0.406	0.15
	1725.5	0.74	0.46	0.728	0.20	0.587	0.32	0.533	0.12	0.484	0.22
	1928.5	0.317	0.07	0.285	0.04	0.213	0.02	0.206	0.01	0.266	0.05

Distance of the positive pressure ventilator 2 from the door opening 1m The inclination angle of the positive pressure ventilator impeller relative to the ground 16°											
		x (mm)									
		91		273		455		637		819	
		AVG	SD	AVG	SD	AVG	SD	AVG	SD	AVG	SD
y (mm)	101.5	1.949	0.39	2.731	0.45	3.03	0.55	2.457	0.68	2.85	1.06
	304.5	3.237	0.88	8.022	1.18	7.365	1.75	5.152	1.00	6.396	0.81
	507.5	4.521	1.62	12.538	1.76	10.25	1.00	9.648	1.18	8.478	1.78
	710.5	6.199	0.97	12.795	1.63	6.93	0.39	14.617	1.83	12.328	1.35
	913.5	6.199	1.43	9.26	1.93	13.379	1.13	13.654	0.78	10.324	2.26
	1116.5	1.032	0.21	2.304	0.71	7.604	3.10	4.188	1.43	3.449	0.98
	1319.5	0.48	0.11	0.606	0.18	0.628	0.17	0.527	0.06	0.745	0.14
	1522.5	0.357	0.10	0.362	0.03	0.424	0.08	0.415	0.06	0.345	0.07
	1725.5	0.438	0.13	0.38	0.06	0.374	0.07	0.397	0.17	0.319	0.06
	1928.5	0.52	0.14	0.509	0.11	0.398	0.09	0.39	0.00	0.403	0.16

Distance of the positive pressure ventilator 2 from the door opening 3 m The inclination angle of the positive pressure ventilator impeller relative to the ground 0°											
		x (mm)									
		91		273		455		637		819	
		AVG	SD	AVG	SD	AVG	SD	AVG	SD	AVG	SD
y (mm)	101.5	7.637	0.37	7.755	0.30	7.884	0.35	7.291	0.45	9.175	0.56
	304.5	7.195	0.37	6.989	0.53	7.764	0.94	8.449	0.28	8.049	0.64
	507.5	5.026	0.56	4.786	0.36	8.16	0.36	8.771	0.24	7.194	0.79
	710.5	2.738	0.55	3.604	0.60	7.147	0.36	7.925	0.55	5.711	0.58
	913.5	2.738	0.43	2.777	0.39	4.595	0.42	5.665	0.65	5.088	0.44
	1116.5	1.439	0.34	1.708	0.49	2.585	0.64	3.102	0.65	2.91	0.56
	1319.5	1.047	0.32	1.117	0.21	1.273	0.27	1.476	0.31	2.164	0.64
	1522.5	0.816	0.23	1.162	0.23	1.09	0.19	1.058	0.12	1.012	0.24
	1725.5	1.131	0.18	0.824	0.19	0.573	0.08	0.556	0.11	0.584	0.15
	1928.5	0.75	0.14	0.985	0.26	0.72	0.20	0.781	0.10	0.44	0.13

Distance of the positive pressure ventilator 2 from the door opening 3 m The inclination angle of the positive pressure ventilator impeller relative to the ground 6°											
		x (mm)									
		91		273		455		637		819	
		AVG	SD	AVG	SD	AVG	SD	AVG	SD	AVG	SD
y (mm)	101.5	6.221	0.53	7.235	0.26	7.122	0.41	7.284	0.50	7.666	0.81
	304.5	6.6	0.37	6.832	0.57	6.57	0.22	6.606	0.67	6.69	0.42
	507.5	6.728	0.33	7.015	0.30	7.229	0.29	6.384	0.52	6.205	0.62
	710.5	4.213	0.62	6.718	0.50	7.504	0.49	6.965	0.64	4.85	0.75
	913.5	4.213	0.82	5.116	0.53	6.943	0.66	7.689	0.68	6.099	0.66
	1116.5	2.423	0.47	4.012	0.92	5.487	0.77	6.871	0.53	4.562	0.61
	1319.5	3.229	0.78	2.932	0.70	4.16	0.70	4.255	0.65	2.792	0.74
	1522.5	0.932	0.14	1.218	0.18	1.466	0.30	1.84	0.31	1.398	0.31
	1725.5	0.527	0.07	0.969	0.27	0.786	0.17	0.865	0.19	0.576	0.14
	1928.5	0.615	0.11	0.713	0.13	0.686	0.12	0.869	0.14	0.477	0.07

Distance of the positive pressure ventilator 2 from the door opening 3 m The inclination angle of the positive pressure ventilator impeller relative to the ground 11°											
		x (mm)									
		91		273		455		637		819	
		AVG	SD	AVG	SD	AVG	SD	AVG	SD	AVG	SD
y (mm)	101.5	3.916	1.17	3.864	0.75	4.417	0.60	6.079	0.92	6.639	0.57
	304.5	5.832	0.53	6.374	0.47	5.997	0.71	5.485	0.74	6.339	0.51
	507.5	5.181	0.55	6.034	0.59	6.329	0.40	5.373	0.26	4.923	0.59
	710.5	5.995	0.48	6.598	0.49	6.173	0.44	5.874	0.42	5.092	0.77
	913.5	5.995	0.61	5.883	0.38	6.025	1.15	6.301	0.69	3.82	0.60
	1116.5	5.122	1.46	6.034	0.84	5.685	0.56	7.958	0.85	4.093	0.83
	1319.5	4.975	0.77	4.06	0.87	6.331	0.72	5.684	1.57	4.245	0.67
	1522.5	4.572	0.44	7.176	0.51	6.307	0.86	6.863	0.73	3.33	0.51
	1725.5	1.911	0.80	2.854	0.62	4.166	0.67	5.364	0.56	2.78	0.59
	1928.5	0.691	0.14	1.253	0.42	2.056	0.17	2.913	0.44	1.737	0.55

Distance of the positive pressure ventilator 2 from the door opening 3 m The inclination angle of the positive pressure ventilator impeller relative to the ground 16°											
		x (mm)									
		91		273		455		637		819	
		AVG	SD	AVG	SD	AVG	SD	AVG	SD	AVG	SD
y (mm)	101.5	1.754	0.35	2.663	0.34	3.547	1.28	2.343	0.43	5.206	0.83
	304.5	2.765	0.64	4.516	1.00	3.885	0.69	4.677	0.62	3.921	0.61
	507.5	4.539	1.04	5.58	0.45	4.727	0.72	4.83	0.56	3.227	0.35
	710.5	3.585	1.24	6.137	0.47	5.536	0.63	4.881	0.59	4.419	0.65
	913.5	3.585	0.57	5.731	0.45	5.158	0.68	5.127	0.83	3.326	0.87
	1116.5	4.272	0.68	5.084	0.59	5.75	0.65	4.3	0.97	3.174	0.87
	1319.5	5.062	0.80	5.2	0.59	5.592	0.62	5.603	0.50	3.79	0.55
	1522.5	4.791	1.43	5.902	0.94	6.319	0.56	7.803	0.31	4.313	0.56
	1725.5	4.503	1.12	3.646	0.52	5.691	0.50	7.281	0.37	3.051	0.56
	1928.5	1.285	0.16	2.389	0.81	3.737	0.31	4.013	0.95	3.297	0.61

Distance of the positive pressure ventilator 2 from the door opening 4 m The inclination angle of the positive pressure ventilator impeller relative to the ground 0°											
		x (mm)									
		91		273		455		637		819	
		AVG	SD	AVG	SD	AVG	SD	AVG	SD	AVG	SD
y (mm)	101.5	7.816	0.31	7.664	0.26	7.418	0.30	7.534	0.14	7.358	0.55
	304.5	6.239	0.42	5.439	0.34	6.369	0.67	7.241	0.32	6.738	0.41
	507.5	4.562	0.36	4.373	0.65	6.039	0.37	6.457	0.38	6.402	0.44
	710.5	2.406	0.49	3.637	0.39	5.547	0.61	6.423	0.34	4.809	0.56
	913.5	2.406	0.31	3.629	0.76	5.111	0.35	5.166	1.01	4.926	0.33
	1116.5	1.994	0.68	2.489	0.44	2.566	0.42	3.356	0.57	3.28	0.40
	1319.5	0.957	0.26	1.591	0.29	1.236	0.19	1.829	0.41	1.957	0.36
	1522.5	0.72	0.13	0.864	0.18	1	0.17	1.447	0.17	1.502	0.20
	1725.5	0.678	0.09	0.704	0.10	0.757	0.17	0.811	0.12	1.208	0.13
	1928.5	0.683	0.11	0.889	0.30	1.427	0.40	0.861	0.17	0.759	0.15

Distance of the positive pressure ventilator 2 from the door opening 4 m The inclination angle of the positive pressure ventilator impeller relative to the ground 6°											
		x (mm)									
		91		273		455		637		819	
		AVG	SD	AVG	SD	AVG	SD	AVG	SD	AVG	SD
y (mm)	101.5	6.514	0.29	6.315	0.24	6.584	0.26	7.061	0.29	6.992	0.36
	304.5	5.022	0.54	5.623	0.33	6.124	0.26	6.217	0.22	6.023	0.55
	507.5	5.378	0.42	5.694	0.54	5.932	0.33	5.521	0.34	6.129	0.40
	710.5	3.231	0.65	5.2	0.86	6.376	0.39	6.211	0.37	5.25	0.64
	913.5	3.231	0.34	5.811	0.17	5.434	0.36	4.846	0.54	3.992	0.38
	1116.5	3.348	0.66	4.556	0.85	5.923	0.35	5.466	1.00	3.77	0.58
	1319.5	2.536	0.48	2.86	0.43	4.232	0.47	4.76	0.78	2.565	0.44
	1522.5	2.435	0.91	4.08	0.60	2.9	0.50	3.84	0.48	2.5	0.44
	1725.5	1.277	0.23	1.445	0.48	1.831	0.35	1.718	0.32	1.582	0.57
	1928.5	1.46	0.38	0.896	0.41	0.928	0.22	1.468	0.32	1.225	0.26

Distance of the positive pressure ventilator 2 from the door opening 4 m The inclination angle of the positive pressure ventilator impeller relative to the ground 11°											
		x (mm)									
		91		273		455		637		819	
		AVG	SD	AVG	SD	AVG	SD	AVG	SD	AVG	SD
y (mm)	101.5	4.931	0.33	5.408	0.59	5.648	0.54	5.59	0.40	5.832	0.26
	304.5	4.406	0.69	4.83	0.58	5.201	0.38	5.644	0.24	5.508	0.50
	507.5	4.217	0.50	4.638	0.52	5.406	0.38	5.318	0.49	5.432	0.46
	710.5	3.39	0.38	5.319	0.40	5.391	0.31	5.019	0.76	4.179	0.42
	913.5	3.39	0.46	5.001	0.78	5.582	0.56	6.049	0.37	4.116	0.50
	1116.5	4.509	0.59	5.213	0.38	5.442	0.41	5.476	0.49	4.269	0.56
	1319.5	4.432	0.73	5.355	0.53	5.956	0.63	5.659	0.59	3.376	0.54
	1522.5	2.873	0.67	4.581	0.59	5.492	0.68	6.663	0.43	2.793	0.75
	1725.5	2.274	0.40	3.939	0.79	4.692	0.49	4.842	0.56	2.872	0.55
	1928.5	1.998	0.48	2.59	0.38	2.863	0.83	4.056	0.35	2.695	0.49

Distance of the positive pressure ventilator 2 from the door opening 4 m The inclination angle of the positive pressure ventilator impeller relative to the ground 16°											
		x (mm)									
		91		273		455		637		819	
		AVG	SD	AVG	SD	AVG	SD	AVG	SD	AVG	SD
y (mm)	101.5	2.309	0.65	3.712	0.51	3.017	0.40	2.498	0.52	3.01	0.28
	304.5	3.408	0.45	4.408	0.26	3.004	0.39	3.348	0.58	2.475	0.61
	507.5	4.3	0.47	5.512	0.34	4.119	0.99	3.386	0.47	2.97	0.43
	710.5	3.368	0.49	5.197	0.47	5.079	0.54	4.806	0.58	4.42	0.49
	913.5	3.368	0.51	5.303	0.29	5.059	0.31	4.93	0.54	4.322	0.78
	1116.5	4.475	0.42	5.152	0.55	5.812	0.28	5.357	0.50	4.057	0.32
	1319.5	3.634	0.58	4.108	0.58	5.316	0.36	5.095	0.43	3.546	0.53
	1522.5	3.384	0.48	4.292	0.53	5.07	0.37	4.732	0.60	3.14	0.55
	1725.5	3.172	0.82	4.192	0.93	5.893	0.54	6.472	0.42	3.558	0.48
	1928.5	3.283	0.40	4.468	0.53	5.686	0.54	6.276	0.43	3.052	0.65

Distance of the positive pressure ventilator 2 from the door opening 5 m The inclination angle of the positive pressure ventilator impeller relative to the ground 0°											
		x (mm)									
		91		273		455		637		819	
		AVG	SD	AVG	SD	AVG	SD	AVG	SD	AVG	SD
y (mm)	101.5	5.641	0.40	5.865	0.44	7.122	0.32	7.012	0.35	7.238	0.39
	304.5	5.212	0.57	5.441	0.44	5.663	0.51	6.376	0.14	6.089	0.44
	507.5	4.7	0.36	5.28	0.49	5.495	0.31	5.392	0.32	3.735	0.51
	710.5	2.426	0.45	4.701	0.34	4.905	0.51	5.119	0.35	3.035	0.36
	913.5	2.426	0.60	2.942	0.59	3.612	0.48	3.961	0.34	3.995	0.56
	1116.5	3.895	0.60	3.233	0.47	2.506	0.61	2.967	0.79	2.05	0.73
	1319.5	2.695	0.39	2.052	0.38	1.671	0.35	1.621	0.29	1.998	0.47
	1522.5	1.418	0.48	1.095	0.27	1.271	0.30	1.504	0.39	1.214	0.19
	1725.5	1.217	0.27	1.137	0.15	1.18	0.21	0.71	0.11	1.357	0.20
	1928.5	0.621	0.11	0.736	0.17	0.74	0.10	0.801	0.12	1.063	0.11

Distance of the positive pressure ventilator 2 from the door opening 5 m The inclination angle of the positive pressure ventilator impeller relative to the ground 6°											
		x (mm)									
		91		273		455		637		819	
		AVG	SD	AVG	SD	AVG	SD	AVG	SD	AVG	SD
y (mm)	101.5	5.797	0.23	5.843	0.26	6.33	0.20	5.95	0.34	6.227	0.17
	304.5	5.102	0.43	5.331	0.30	5.554	0.32	5.559	0.20	5.438	0.70
	507.5	4.471	0.73	5.169	0.44	5.114	0.33	5.387	0.32	4.832	0.47
	710.5	3.673	0.50	5.17	0.45	4.766	0.36	4.592	0.48	3.237	0.32
	913.5	3.673	0.38	4.394	0.51	5.232	0.32	5.11	0.43	4.375	0.49
	1116.5	3.022	0.49	4.061	0.32	4.72	0.29	4.989	0.27	3.679	0.56
	1319.5	2.928	0.69	3.784	0.75	3.736	0.35	4.247	0.39	2.352	0.37
	1522.5	3.475	0.45	3.297	0.41	4.034	0.59	3.769	0.57	1.739	0.43
	1725.5	3.609	0.59	3.416	0.45	2.357	0.42	2.243	0.51	1.532	0.32
	1928.5	1.274	0.38	1.326	0.33	1.664	0.48	2.233	0.49	1.544	0.37

Distance of the positive pressure ventilator 2 from the door opening 5 m The inclination angle of the positive pressure ventilator impeller relative to the ground 11°											
		x (mm)									
		91		273		455		637		819	
		AVG	SD	AVG	SD	AVG	SD	AVG	SD	AVG	SD
y (mm)	101.5	4.32	0.62	4.68	0.32	5.356	0.37	5.12	0.38	4.979	0.36
	304.5	4.677	0.21	4.681	0.41	4.858	0.27	5.103	0.37	4.155	0.68
	507.5	4.261	0.25	4.351	0.57	5.045	0.31	4.774	0.28	4.747	0.42
	710.5	3.614	0.39	4.626	0.53	4.025	0.41	4.894	0.33	4.374	0.21
	913.5	3.614	0.60	4.408	0.54	4.922	0.40	4.991	0.43	4.58	0.44
	1116.5	3.576	0.62	4.378	0.69	4.871	0.33	5.058	0.60	3.744	0.54
	1319.5	3.138	0.54	4.416	0.43	4.974	0.33	4.841	0.43	3.25	0.48
	1522.5	4.439	0.42	4.833	0.38	4.764	0.41	4.392	0.39	3.58	0.65
	1725.5	3.768	0.49	4.232	0.54	4.428	0.49	4.12	0.39	2.636	0.35
	1928.5	3.713	0.49	4.142	0.48	4.604	0.60	4.74	0.55	3.149	0.54

Distance of the positive pressure ventilator 2 from the door opening 5 m The inclination angle of the positive pressure ventilator impeller relative to the ground 16°											
		x (mm)									
		91		273		455		637		819	
		AVG	SD	AVG	SD	AVG	SD	AVG	SD	AVG	SD
y (mm)	101.5	2.368	0.64	2.77	0.75	4.426	0.75	3.605	0.46	3.949	0.61
	304.5	2.808	0.41	2.741	1.01	3.5	0.55	3.848	0.76	3.471	0.34
	507.5	3.279	0.77	3.242	0.75	4.379	0.39	4.108	0.50	2.749	0.54
	710.5	3.265	0.54	4.069	0.37	4.199	0.68	3.935	0.44	3.539	0.66
	913.5	3.265	0.64	3.645	0.88	4.201	0.79	4.331	0.58	3.823	0.34
	1116.5	3.984	0.59	5.039	0.54	4.394	0.54	4.51	0.29	4.079	0.46
	1319.5	3.722	0.49	4.495	0.56	4.773	0.34	4.723	0.39	3.521	0.65
	1522.5	2.167	0.51	3.238	0.45	3.643	0.74	5.259	0.50	3.333	0.80
	1725.5	2.897	0.69	4.019	0.75	5.097	0.27	4.348	0.72	3.7	0.47
	1928.5	4.643	0.54	4.261	0.80	5.133	0.66	5.542	0.51	3.591	0.75

Distance of the positive pressure ventilator 2 from the door opening 7 m The inclination angle of the positive pressure ventilator impeller relative to the ground 0°											
		x (mm)									
		91		273		455		637		819	
		AVG	SD	AVG	SD	AVG	SD	AVG	SD	AVG	SD
y (mm)	101.5	4.131	0.15	4.726	0.40	6.108	0.32	5.642	0.34	6.029	0.48
	304.5	4.42	0.53	5.246	0.21	5.272	0.21	5.411	0.41	5.713	0.58
	507.5	4.041	0.44	4.259	0.44	3.978	0.27	4.941	0.26	4.414	0.32
	710.5	1.774	0.45	3.894	0.37	3.915	0.45	4.181	0.38	3.554	0.38
	913.5	1.774	0.37	3.514	0.30	3.242	0.45	3.693	0.66	3.458	0.41
	1116.5	2.819	0.49	2.676	0.69	2.397	0.33	3.167	0.47	2.887	0.46
	1319.5	1.364	0.43	1.65	0.34	1.985	0.36	2.326	0.57	1.875	0.44
	1522.5	1.237	0.24	1.733	0.28	1.317	0.41	1.547	0.30	1.158	0.23
	1725.5	0.749	0.21	0.871	0.17	0.838	0.13	0.959	0.21	1.024	0.27
	1928.5	0.741	0.16	0.708	0.09	0.946	0.15	0.738	0.16	0.737	0.18

Distance of the positive pressure ventilator 2 from the door opening 7 m The inclination angle of the positive pressure ventilator impeller relative to the ground 6°											
		x (mm)									
		91		273		455		637		819	
		AVG	SD	AVG	SD	AVG	SD	AVG	SD	AVG	SD
y (mm)	101.5	4.105	0.52	5.119	0.34	5.026	0.33	5.949	0.35	6.341	0.34
	304.5	4.616	0.46	4.324	0.44	4.869	0.53	5.25	0.40	5.622	0.51
	507.5	4.383	0.42	4.378	0.42	4.428	0.33	4.905	0.18	4.385	0.51
	710.5	3.08	0.34	3.615	0.51	4.028	0.39	4.575	0.33	3.884	0.43
	913.5	3.08	0.42	3.856	0.40	3.92	0.42	4.145	0.34	4.22	0.11
	1116.5	2.374	0.62	3.751	0.37	4.318	0.44	3.37	0.31	3.627	0.33
	1319.5	3.747	0.54	3.898	0.29	3.457	0.43	3.197	0.57	2.937	0.40
	1522.5	3.438	0.36	3.163	0.47	2.534	0.86	3.113	0.51	2.625	0.24
	1725.5	1.88	0.57	2.6	0.56	2.007	0.36	2.596	0.86	2.02	0.26
	1928.5	2.938	0.40	2.385	0.57	1.806	0.79	1.99	0.47	2.348	0.55

Distance of the positive pressure ventilator 2 from the door opening 7 m The inclination angle of the positive pressure ventilator impeller relative to the ground 11°											
		x (mm)									
		91		273		455		637		819	
		AVG	SD	AVG	SD	AVG	SD	AVG	SD	AVG	SD
y (mm)	101.5	4.305	0.52	3.978	0.42	4.233	0.47	4.478	0.37	4.485	0.40
	304.5	2.94	0.37	3.521	0.36	4.292	0.41	4.441	0.48	4.668	0.53
	507.5	2.851	0.36	3.293	0.64	4.069	0.29	4.197	0.32	3.521	0.67
	710.5	3.482	0.28	3.639	0.51	3.651	0.44	3.926	0.48	4.239	0.52
	913.5	3.482	0.50	3.715	0.32	3.651	0.46	3.693	0.57	3.277	0.34
	1116.5	3.462	0.49	3.421	0.30	3.279	0.45	3.384	0.49	3.546	0.50
	1319.5	3.049	0.46	2.849	0.74	3.518	0.27	3.64	0.36	3.161	0.36
	1522.5	3.996	0.40	3.682	0.51	3.325	0.28	3.529	0.56	2.426	0.46
	1725.5	3.743	0.31	3.57	0.53	3.502	0.67	3.071	0.56	3.039	0.40
	1928.5	3.906	0.49	3.387	0.37	3.266	0.59	3.294	0.43	2.732	0.55

Distance of the positive pressure ventilator 2 from the door opening 7 m The inclination angle of the positive pressure ventilator impeller relative to the ground 16°											
		x (mm)									
		91		273		455		637		819	
		AVG	SD	AVG	SD	AVG	SD	AVG	SD	AVG	SD
y (mm)	101.5	3.85	0.28	3.798	0.28	4.047	0.53	3.594	0.44	3.97	0.34
	304.5	3.367	0.34	3.54	0.32	3.319	0.53	3.556	0.26	3.649	0.53
	507.5	3.059	0.61	2.892	0.38	3.315	0.52	3.674	0.51	3.449	0.49
	710.5	2.851	0.25	2.903	0.42	3.324	0.47	3.506	0.68	3.128	0.43
	913.5	2.851	0.42	2.272	0.68	3.015	0.49	3.043	0.45	2.081	0.48
	1116.5	1.985	0.33	2.247	0.52	3.261	0.59	2.56	0.60	1.594	0.33
	1319.5	2.554	0.66	2.526	0.53	2.696	0.48	3.237	0.70	1.709	0.48
	1522.5	2.538	0.64	2.194	0.50	3.352	0.57	3.327	0.43	1.126	0.36
	1725.5	2.528	0.61	2.434	0.48	3.093	0.71	3.224	0.61	1.476	0.56
	1928.5	2.467	0.57	3.095	0.76	2.928	0.63	3.2	0.63	2.477	0.64

AVG—arithmetic mean of the flow rate, SD—standard deviation taken as the measurement error.

**Table 4:** Air flow velocity in the door opening for fan 3, where: x—position of the measuring point in relation to the x-axis and, y—position of the measuring point in relation to the y-axis.

Distance of the positive pressure ventilator 3 from the door opening 1m The inclination angle of the positive pressure ventilator impeller relative to the ground 0°											
		x (mm)									
		91		273		455		637		819	
		AVG	SD	AVG	SD	AVG	SD	AVG	SD	AVG	SD
y (mm)	101.5	0.997	0.13	8.263	0.54	24.905	0.58	8.673	0.89	1.243	0.11
	304.5	1.377	0.26	19.29	0.23	27.268	0.15	17.739	0.44	1.326	0.19
	507.5	0.717	0.07	4.788	0.68	12.923	0.57	4.859	0.36	0.9	0.19
	710.5	0.845	0.17	1.023	0.25	1.147	0.28	0.859	0.22	0.546	0.15
	913.5	0.845	0.32	0.555	0.20	0.408	0.09	0.458	0.10	0.425	0.20
	1116.5	0.882	0.33	0.505	0.16	0.553	0.20	0.242	0.03	0.443	0.19
	1319.5	0.522	0.15	0.385	0.10	0.725	0.27	1.202	0.39	0.415	0.23
	1522.5	0.216	0.02	0.234	0.01	0.769	0.32	0.29	0.08	0.526	0.27
	1725.5	0.6	0.27	0.202	0.02	0.235	0.02	0.208	0.02	0.289	0.09
	1928.5	0.202	0.02	0.188	0.01	0.157	0.04	0.197	0.02	0.223	0.02

Distance of the positive pressure ventilator 3 from the door opening 1m The inclination angle of the positive pressure ventilator impeller relative to the ground 6°											
		x (mm)									
		91		273		455		637		819	
		AVG	SD	AVG	SD	AVG	SD	AVG	SD	AVG	SD
y (mm)	101.5	1.467	0.19	1.982	0.13	4.968	0.66	2.309	0.46	1	0.27
	304.5	1.566	0.10	13.65	0.99	28.754	0.30	13.876	0.91	1.221	0.18
	507.5	1.786	0.14	14.949	0.92	29.482	0.25	18.741	0.86	2.036	0.30
	710.5	0.708	0.30	2.593	0.45	5.998	0.55	2.117	0.18	0.657	0.06
	913.5	0.708	0.13	1.292	0.29	0.661	0.14	0.597	0.27	0.514	0.28
	1116.5	0.473	0.17	0.454	0.31	0.586	0.31	0.494	0.29	0.408	0.09
	1319.5	0.582	0.23	0.575	0.27	0.637	0.44	1.195	0.48	0.395	0.21
	1522.5	0.413	0.04	0.356	0.07	0.649	0.14	0.385	0.08	0.422	0.14
	1725.5	0.244	0.02	0.276	0.04	0.223	0.02	0.268	0.05	0.292	0.04
	1928.5	0.331	0.04	0.548	0.20	0.321	0.17	0.298	0.08	0.443	0.12

Distance of the positive pressure ventilator 3 from the door opening 1m The inclination angle of the positive pressure ventilator impeller relative to the ground 12°											
		x (mm)									
		91		273		455		637		819	
		AVG	SD	AVG	SD	AVG	SD	AVG	SD	AVG	SD
y (mm)	101.5	1.357	0.08	1.629	0.15	0.991	0.26	0.692	0.12	0.893	0.22
	304.5	0.935	0.26	3.014	0.26	12.256	0.76	3.586	0.49	1.334	0.33
	507.5	1.307	0.10	18.293	0.58	28.862	0.30	18.677	0.48	1.553	0.12
	710.5	0.741	0.18	13.427	0.73	28.127	0.35	11.324	0.52	1.268	0.35
	913.5	0.741	0.15	1.306	0.27	3.405	0.65	1.553	0.22	0.65	0.08
	1116.5	0.502	0.20	0.669	0.20	0.688	0.16	0.644	0.20	0.726	0.16
	1319.5	0.383	0.12	0.426	0.15	0.602	0.20	0.68	0.18	0.409	0.10
	1522.5	0.575	0.19	0.354	0.05	0.446	0.13	0.473	0.12	0.538	0.21
	1725.5	0.352	0.11	0.323	0.07	0.401	0.06	0.285	0.05	0.363	0.10
	1928.5	0.429	0.15	0.288	0.07	0.556	0.15	0.401	0.10	0.356	0.10

Distance of the positive pressure ventilator 3 from the door opening 1m The inclination angle of the positive pressure ventilator impeller relative to the ground 18°											
		x (mm)									
		91		273		455		637		819	
		AVG	SD	AVG	SD	AVG	SD	AVG	SD	AVG	SD
y (mm)	101.5	0.37	0.04	1.129	0.58	1.689	0.27	1.737	0.13	0.832	0.21
	304.5	1.145	0.33	1.626	0.32	1.711	0.34	1.312	0.35	0.79	0.29
	507.5	1.078	0.22	6.808	0.56	20.328	0.48	11.635	1.56	2.466	0.38
	710.5	0.85	0.18	20.031	0.64	27.992	0.27	17.875	1.19	1.699	0.51
	913.5	0.85	0.09	8.844	1.13	22.129	0.59	10.601	0.85	1.044	0.20
	1116.5	0.798	0.17	0.934	0.12	1.708	0.41	1.365	0.20	0.768	0.14
	1319.5	0.553	0.13	0.551	0.11	0.899	0.23	0.448	0.06	0.642	0.17
	1522.5	0.468	0.12	0.512	0.17	0.61	0.20	0.577	0.22	0.328	0.08
	1725.5	0.641	0.34	0.605	0.19	0.427	0.21	0.631	0.16	0.478	0.14
	1928.5	0.343	0.07	0.371	0.05	0.262	0.06	0.332	0.07	0.416	0.11

Distance of the positive pressure ventilator 3 from the door opening 3 m The inclination angle of the positive pressure ventilator impeller relative to the ground 0°											
		x (mm)									
		91		273		455		637		819	
		AVG	SD	AVG	SD	AVG	SD	AVG	SD	AVG	SD
y (mm)	101.5	10.634	0.67	15.31	0.63	17.996	0.52	14.44	0.56	8.744	0.71
	304.5	7.208	0.52	14.951	0.79	18.477	0.37	14.358	0.71	6.673	0.59
	507.5	4.96	0.79	9.593	1.09	12.577	0.91	10.118	0.95	5.482	0.56
	710.5	1.125	0.68	3.883	0.57	4.185	0.76	4.312	0.99	1.778	0.50
	913.5	1.125	0.16	0.897	0.25	0.88	0.27	0.839	0.14	0.935	0.14
	1116.5	0.75	0.14	0.62	0.24	1.203	0.35	0.781	0.31	0.931	0.18
	1319.5	0.371	0.08	1.022	0.41	0.702	0.34	0.716	0.19	0.569	0.24
	1522.5	0.398	0.09	0.826	0.22	0.925	0.23	0.788	0.26	0.496	0.15
	1725.5	0.367	0.11	0.305	0.08	0.429	0.13	0.458	0.10	0.424	0.17
	1928.5	0.403	0.16	0.289	0.05	0.274	0.04	0.233	0.03	0.248	0.05

Distance of the positive pressure ventilator 3 from the door opening 3 m The inclination angle of the positive pressure ventilator impeller relative to the ground 6°											
		x (mm)									
		91		273		455		637		819	
		AVG	SD	AVG	SD	AVG	SD	AVG	SD	AVG	SD
y (mm)	101.5	3.244	0.65	5.879	0.54	7.119	0.56	3.728	0.69	1.628	0.45
	304.5	5.254	0.95	9.752	1.28	11.23	0.65	7.25	0.75	2.717	0.40
	507.5	6.589	0.55	13.102	1.13	16.919	0.62	12.125	0.78	5.673	0.51
	710.5	5.976	0.60	15.221	0.55	17.199	0.54	11.281	1.04	5.432	0.74
	913.5	5.976	0.41	10.516	1.34	11.249	0.99	7.996	0.70	2.231	0.50
	1116.5	3.366	0.93	4.243	0.69	4.219	0.92	2.472	0.36	1.351	0.19
	1319.5	1.169	0.49	1.505	0.49	1.191	0.31	1.026	0.21	0.525	0.07
	1522.5	0.688	0.16	0.924	0.23	0.694	0.26	0.547	0.16	0.295	0.04
	1725.5	1.044	0.20	0.347	0.06	0.33	0.13	0.826	0.25	0.546	0.22
	1928.5	0.694	0.24	0.448	0.17	0.356	0.08	0.783	0.26	0.354	0.07

Distance of the positive pressure ventilator 3 from the door opening 3 m The inclination angle of the positive pressure ventilator impeller relative to the ground 12°											
		x (mm)									
		91		273		455		637		819	
		AVG	SD	AVG	SD	AVG	SD	AVG	SD	AVG	SD
y (mm)	101.5	1.537	0.31	1.202	0.56	0.96	0.38	0.736	0.30	0.707	0.27
	304.5	2.009	0.66	2.307	0.57	1.745	0.51	1.247	0.26	0.851	0.22
	507.5	4.302	0.75	5.875	1.18	4.644	0.87	5.467	0.95	3.102	0.97
	710.5	6.674	0.68	10.514	0.84	12.715	0.92	9.342	0.83	6.622	0.81
	913.5	6.674	1.16	14.201	0.75	17.899	0.32	13.535	0.43	7.055	0.90
	1116.5	6.116	0.94	13.777	0.86	17.117	0.45	11.538	1.06	6.181	0.65
	1319.5	4.257	0.84	9.092	0.43	10.092	0.80	8.5	0.66	5.293	0.79
	1522.5	3.13	0.53	4.659	0.75	4.205	0.61	3.793	0.78	1.85	0.48
	1725.5	1.311	0.20	1.378	0.37	1.106	0.41	0.762	0.12	0.787	0.11
	1928.5	0.397	0.09	0.615	0.19	0.383	0.06	0.406	0.11	0.391	0.07

Distance of the positive pressure ventilator 3 from the door opening 3 m The inclination angle of the positive pressure ventilator impeller relative to the ground 18°											
		x (mm)									
		91		273		455		637		819	
		AVG	SD	AVG	SD	AVG	SD	AVG	SD	AVG	SD
y (mm)	101.5	0.521	0.19	1.079	0.36	1.36	0.22	1.141	0.17	0.663	0.13
	304.5	1.078	0.22	1.201	0.31	0.994	0.16	0.463	0.10	1.089	0.29
	507.5	0.687	0.19	0.768	0.20	0.948	0.19	1.318	0.34	0.62	0.09
	710.5	3.35	0.46	2.459	0.48	3.057	0.84	1.429	0.33	1.291	0.31
	913.5	3.35	0.41	5.198	1.25	7.86	0.63	5.322	0.94	3.153	0.76
	1116.5	6.74	0.76	11.448	0.85	12.458	0.62	9.967	0.71	6.087	0.44
	1319.5	8.199	0.92	13.773	1.16	17.715	0.42	13.8	0.55	7.53	0.63
	1522.5	6.962	0.75	13.811	0.57	16.348	0.61	12.391	0.91	7.113	0.99
	1725.5	4.741	0.60	9.089	0.79	11.342	0.76	9.835	0.43	5.671	0.70
	1928.5	3.03	0.49	5.161	1.11	5.504	0.57	4.238	0.68	3.584	0.54

Distance of the positive pressure ventilator 3 from the door opening 4 m The inclination angle of the positive pressure ventilator impeller relative to the ground 0°											
		x (mm)									
		91		273		455		637		819	
		AVG	SD	AVG	SD	AVG	SD	AVG	SD	AVG	SD
y (mm)	101.5	10.992	0.93	14.189	0.28	15.188	0.35	13.118	0.34	9.802	0.49
	304.5	8.253	0.77	12.823	0.37	14.881	0.62	11.407	0.63	8.223	0.55
	507.5	7.161	0.70	10.007	0.61	10.542	0.76	11.084	0.59	6.825	0.70
	710.5	1.762	0.52	6.397	0.94	6.548	0.96	4.861	0.45	4.031	0.43
	913.5	1.762	0.49	2.54	0.53	2.285	0.49	2.241	0.58	1.491	0.36
	1116.5	0.932	0.22	1.047	0.20	1.29	0.57	1.926	0.53	2.125	0.43
	1319.5	0.766	0.20	2.264	0.43	0.585	0.13	1.338	0.62	0.445	0.07
	1522.5	1.568	0.35	1.178	0.30	0.851	0.70	1.031	0.39	0.417	0.12
	1725.5	0.61	0.26	0.487	0.10	0.719	0.39	1.373	0.27	0.956	0.30
	1928.5	0.737	0.33	0.336	0.06	1.068	0.47	0.611	0.21	0.59	0.14

Distance of the positive pressure ventilator 3 from the door opening 4 m The inclination angle of the positive pressure ventilator impeller relative to the ground 6°											
		x (mm)									
		91		273		455		637		819	
		AVG	SD	AVG	SD	AVG	SD	AVG	SD	AVG	SD
y (mm)	101.5	5.35	0.75	6.016	0.62	5.95	0.68	4.569	1.12	2.945	0.70
	304.5	5.831	0.92	7.882	0.63	8.358	0.67	7.197	0.90	3.674	0.48
	507.5	7.555	0.69	10.988	0.58	12.027	0.57	9.985	0.84	5.793	1.01
	710.5	7.173	0.67	12.053	1.06	13.495	0.49	10.747	0.83	6.935	0.90
	913.5	7.173	0.77	10.084	1.02	11.655	0.61	8.862	0.79	5.316	1.19
	1116.5	6.187	0.91	9.35	0.80	7.772	0.86	6.672	1.44	3.803	0.74
	1319.5	3.888	1.07	4.328	0.80	4.982	0.87	2.035	0.51	1.739	0.29
	1522.5	1.494	0.48	1.809	0.49	1.643	0.39	1.361	0.38	1.083	0.27
	1725.5	0.937	0.18	0.532	0.09	0.599	0.16	0.907	0.35	1.396	0.34
	1928.5	0.575	0.12	0.435	0.10	0.522	0.15	0.619	0.20	0.813	0.21

Distance of the positive pressure ventilator 3 from the door opening 4 m The inclination angle of the positive pressure ventilator impeller relative to the ground 12°											
		x (mm)									
		91		273		455		637		819	
		AVG	SD	AVG	SD	AVG	SD	AVG	SD	AVG	SD
y (mm)	101.5	1.29	0.19	1.819	0.26	1.562	0.16	1.12	0.13	1.063	0.23
	304.5	1.098	0.20	1.505	0.38	1.328	0.29	1.366	0.17	1.558	0.50
	507.5	3.295	0.66	4.094	0.76	3.271	0.73	2.583	0.60	2.052	0.60
	710.5	7.068	0.60	6.435	0.85	5.653	0.46	5.837	0.79	3.813	0.83
	913.5	7.068	0.68	10.329	0.53	10.252	0.70	9.065	0.56	6.601	0.70
	1116.5	8.289	0.64	12.065	0.79	13.707	0.39	11.279	0.44	7.034	0.64
	1319.5	7.248	0.88	11.681	0.70	13.569	0.41	10.771	0.69	7.515	0.90
	1522.5	6.678	0.57	10.237	0.56	10.529	0.61	8.833	0.66	5.818	0.55
	1725.5	5.011	0.54	5.76	0.54	6.7	0.72	4.483	0.58	4.095	0.55
	1928.5	2.239	0.45	3.509	0.44	3.518	0.74	3.029	0.60	3.023	0.53

Distance of the positive pressure ventilator 3 from the door opening 4 m The inclination angle of the positive pressure ventilator impeller relative to the ground 18°											
		x (mm)									
		91		273		455		637		819	
		AVG	SD	AVG	SD	AVG	SD	AVG	SD	AVG	SD
y (mm)	101.5	0.796	0.42	1.261	0.17	1.047	0.45	0.561	0.19	0.388	0.09
	304.5	1.073	0.23	0.909	0.24	1.008	0.33	0.789	0.39	0.41	0.06
	507.5	0.779	0.23	0.959	0.21	0.978	0.37	1.019	0.29	0.866	0.19
	710.5	0.798	0.18	0.73	0.19	0.956	0.17	0.817	0.25	1.306	0.43
	913.5	0.798	0.26	1.479	0.42	1.153	0.30	1.473	0.29	2.919	0.51
	1116.5	3.026	0.70	4.392	0.66	4.218	0.86	4.708	0.55	4.352	0.50
	1319.5	4.864	0.91	7.062	0.74	8.299	0.87	8.419	0.81	6.404	0.46
	1522.5	7.7	0.45	10.078	0.67	11.76	0.73	11.184	0.49	7.898	0.58
	1725.5	6.987	0.48	11.426	0.55	13.401	0.51	12.546	0.53	8.697	0.62
	1928.5	7.811	0.38	10.752	0.54	11.929	0.55	11.226	0.66	9.008	0.67

Distance of the positive pressure ventilator 3 from the door opening 5 m The inclination angle of the positive pressure ventilator impeller relative to the ground 0°											
		x (mm)									
		91		273		455		637		819	
		AVG	SD	AVG	SD	AVG	SD	AVG	SD	AVG	SD
y (mm)	101.5	10.443	0.40	13.073	0.45	13.931	0.49	12.3	0.41	9.888	0.78
	304.5	9.119	0.55	11.367	0.45	12.157	0.59	11.011	0.49	8.554	0.74
	507.5	7.062	0.52	9.043	0.77	9.78	0.55	8.026	0.69	7.079	0.36
	710.5	3.188	0.63	6.644	0.74	6.974	0.75	5.959	0.77	5.089	0.59
	913.5	3.188	0.41	3.059	0.65	3.699	0.62	3.513	0.32	3.231	0.65
	1116.5	1.934	0.39	1.482	0.49	1.704	0.51	1.399	0.25	1.708	0.50
	1319.5	1.353	0.37	0.864	0.18	1.097	0.33	1.335	0.23	0.764	0.14
	1522.5	0.682	0.12	0.975	0.23	0.583	0.09	0.948	0.23	0.812	0.17
	1725.5	0.775	0.15	0.617	0.06	1.655	0.28	0.563	0.13	1.225	0.28
	1928.5	0.979	0.19	1.087	0.24	0.821	0.14	0.577	0.12	0.505	0.10

Distance of the positive pressure ventilator 3 from the door opening 5 m The inclination angle of the positive pressure ventilator impeller relative to the ground 6°											
		x (mm)									
		91		273		455		637		819	
		AVG	SD	AVG	SD	AVG	SD	AVG	SD	AVG	SD
y (mm)	101.5	5.033	0.58	6.54	0.51	6.19	0.32	5.227	0.72	3.916	0.65
	304.5	6.814	0.59	7.575	0.46	8.182	0.74	6.676	1.16	4.319	0.62
	507.5	6.797	0.67	8.507	0.54	9.737	0.31	8.41	0.52	5.785	0.67
	710.5	7.292	0.68	10.092	0.87	10.728	0.40	8.982	0.62	6.315	1.11
	913.5	7.292	0.67	9.687	0.70	10.561	0.32	9.095	0.96	6.705	0.72
	1116.5	6.694	0.63	8.449	0.55	8.17	1.56	8.048	0.93	5.342	1.21
	1319.5	5.365	0.65	6.232	0.47	6.123	1.14	5.045	0.60	3.4	0.42
	1522.5	3.49	0.69	4.492	0.73	4.246	0.45	2.53	0.94	1.756	0.55
	1725.5	2.199	0.36	1.724	0.24	1.584	0.54	1.254	0.14	1.203	0.29
	1928.5	1.1	0.23	1.273	0.26	0.85	0.21	0.898	0.28	0.612	0.26

Distance of the positive pressure ventilator 3 from the door opening 5 m The inclination angle of the positive pressure ventilator impeller relative to the ground 12°											
		x (mm)									
		91		273		455		637		819	
		AVG	SD	AVG	SD	AVG	SD	AVG	SD	AVG	SD
y (mm)	101.5	0.926	0.30	1.149	0.22	1.204	0.21	0.742	0.18	1.056	0.13
	304.5	1.826	0.21	1.188	0.22	1.475	0.37	1.483	0.40	1.527	0.37
	507.5	2.484	0.38	2.136	0.55	3.321	0.44	3.018	0.57	3.059	0.53
	710.5	5.251	0.34	4.562	0.48	4.64	1.04	4.776	0.77	3.943	0.57
	913.5	5.251	0.97	6.999	0.56	7.356	0.54	6.388	0.53	5.118	0.55
	1116.5	6.641	0.59	8.244	0.78	9.615	0.65	8.223	0.54	5.918	0.86
	1319.5	6.3	0.80	9.509	0.66	11.147	0.46	9.965	0.48	6.384	0.76
	1522.5	6.84	0.75	10.228	0.43	11.1	0.37	10.568	0.46	7.44	0.60
	1725.5	6.669	0.82	8.169	0.48	9.358	0.56	8.702	0.56	5.587	0.79
	1928.5	5.338	0.77	5.97	0.52	6.922	0.67	6.528	0.54	5.287	0.60

Distance of the positive pressure ventilator 3 from the door opening 5 m The inclination angle of the positive pressure ventilator impeller relative to the ground 18°											
		x (mm)									
		91		273		455		637		819	
		AVG	SD	AVG	SD	AVG	SD	AVG	SD	AVG	SD
y (mm)	101.5	0.559	0.11	0.375	0.10	0.418	0.09	0.677	0.22	0.816	0.21
	304.5	0.607	0.16	0.945	0.19	0.67	0.20	1.22	0.12	0.962	0.21
	507.5	0.782	0.25	0.975	0.19	0.968	0.20	0.976	0.17	0.953	0.19
	710.5	0.927	0.29	1.521	0.46	0.7	0.20	0.695	0.17	0.709	0.36
	913.5	0.927	0.21	0.81	0.15	1.039	0.24	0.856	0.15	1.144	0.42
	1116.5	1.839	0.27	1.902	0.65	2.971	0.66	2.413	0.55	3.052	0.79
	1319.5	3.187	0.74	3.742	0.81	4.151	1.00	4.707	0.44	4.797	0.56
	1522.5	5.074	0.63	6.273	0.55	6.568	0.71	6.97	0.55	5.727	0.69
	1725.5	7.086	0.54	8.448	0.55	9.067	0.88	9.121	0.41	7.547	0.34
	1928.5	9.236	0.34	10.668	0.84	11.256	0.55	10.691	0.64	9.441	0.52

Distance of the positive pressure ventilator 3 from the door opening 7 m The inclination angle of the positive pressure ventilator impeller relative to the ground 0°											
		x (mm)									
		91		273		455		637		819	
		AVG	SD	AVG	SD	AVG	SD	AVG	SD	AVG	SD
y (mm)	101.5	9.305	0.37	10.461	0.40	10.646	0.61	10.095	0.48	9.579	0.79
	304.5	8.373	0.54	9.137	0.50	10.021	0.27	9.496	0.62	8.7	0.65
	507.5	6.563	0.52	8.038	0.47	8.137	0.61	7.963	0.67	7.384	0.27
	710.5	4.07	0.57	6.163	0.68	6.454	0.65	6.491	0.55	6.101	0.85
	913.5	4.07	0.75	4.714	0.54	4.552	0.82	4.651	0.30	4.875	0.68
	1116.5	2.379	0.55	3.204	0.66	2.901	0.66	3.151	0.43	3.687	0.29
	1319.5	1.33	0.29	2.036	0.47	1.559	0.28	2.135	0.33	2.969	0.49
	1522.5	1.132	0.15	1.194	0.11	1.149	0.18	1.47	0.34	2.131	0.66
	1725.5	0.749	0.18	1	0.15	0.869	0.18	0.909	0.28	1.031	0.26
	1928.5	0.996	0.19	0.809	0.19	1.093	0.33	1.077	0.27	0.959	0.22

Distance of the positive pressure ventilator 3 from the door opening 7 m The inclination angle of the positive pressure ventilator impeller relative to the ground 6°											
		x (mm)									
		91		273		455		637		819	
		AVG	SD	AVG	SD	AVG	SD	AVG	SD	AVG	SD
y (mm)	101.5	4.229	0.56	5.66	0.25	5.263	0.42	4.529	0.62	3.039	0.80
	304.5	5.81	0.56	5.798	0.61	5.458	0.78	4.353	0.74	3.366	0.50
	507.5	5.932	0.35	6.643	0.37	6.567	0.64	5.283	0.49	4.012	0.83
	710.5	6.553	0.44	7.416	0.47	7.469	0.53	6.278	0.41	4.662	0.65
	913.5	6.553	0.38	7.725	0.69	7.976	0.43	6.967	0.56	5.237	0.83
	1116.5	6.002	0.66	7.293	0.49	8.194	0.54	7.387	0.71	5.054	0.44
	1319.5	5.391	0.60	6.963	0.64	7.824	0.63	7.418	0.50	5.588	0.57
	1522.5	5.188	0.62	6.149	0.49	5.927	0.59	6.508	0.56	4.711	0.61
	1725.5	3.859	0.48	4.661	0.64	5.484	0.53	5.288	0.55	4.312	0.65
	1928.5	2.78	0.59	3.493	0.52	4.172	0.71	3.814	0.64	3.401	0.46

Distance of the positive pressure ventilator 3 from the door opening 7 m The inclination angle of the positive pressure ventilator impeller relative to the ground 12°											
		x (mm)									
		91		273		455		637		819	
		AVG	SD	AVG	SD	AVG	SD	AVG	SD	AVG	SD
y (mm)	102	0.857	0.32	0.91	0.23	0.839	0.20	1.032	0.21	0.964	0.35
	305	1.426	0.08	0.884	0.14	0.954	0.19	1.11	0.26	0.997	0.29
	508	0.762	0.22	1.123	0.17	1.545	0.39	1.122	0.36	1.375	0.31
	711	2.125	0.46	1.94	0.73	1.618	0.51	2.403	0.55	2.748	0.59
	914	2.125	0.66	2.656	0.53	3.032	0.70	3.677	0.48	3.948	0.44
	1117	4.017	0.57	3.926	0.48	4.523	0.51	5.034	0.68	3.955	0.60
	1320	4.925	0.49	5.288	0.34	5.625	0.63	6.121	0.66	4.64	0.55
	1523	6.257	0.50	6.435	0.65	6.793	0.44	6.932	0.46	5.463	0.54
	1726	5.82	0.72	7.258	0.34	7.817	0.42	7.757	0.54	5.837	0.42
	1929	6.82	0.80	7.3	0.48	8.025	0.78	8.037	0.44	7.517	0.41

Distance of the positive pressure ventilator 3 from the door opening 7 m The inclination angle of the positive pressure ventilator impeller relative to the ground 18°											
		x (mm)									
		91		273		455		637		819	
		AVG	SD	AVG	SD	AVG	SD	AVG	SD	AVG	SD
y (mm)	101.5	0.814	0.28	1.256	0.22	1.404	0.24	1.302	0.29	1.207	0.35
	304.5	0.965	0.18	0.682	0.18	0.967	0.26	1.046	0.23	0.819	0.26
	507.5	0.535	0.12	0.64	0.24	0.648	0.18	0.563	0.17	0.632	0.15
	710.5	0.958	0.17	0.441	0.08	0.458	0.17	0.494	0.12	0.518	0.11
	913.5	0.958	0.24	0.579	0.12	0.486	0.10	0.516	0.16	0.489	0.17
	1116.5	1.283	0.39	0.876	0.18	1.068	0.29	0.819	0.27	0.576	0.26
	1319.5	1.98	0.34	1.484	0.39	1.426	0.50	0.98	0.25	1.545	0.38
	1522.5	2.47	0.39	2.047	0.61	2.47	0.46	2.324	0.36	2.196	0.39
	1725.5	2.737	0.61	3.652	0.57	3.31	0.37	3.281	0.63	2.896	0.56
	1928.5	2.292	0.31	1.605	0.38	1.97	0.49	2.055	0.34	2.382	0.39

AVG—arithmetic mean of the flow rate, SD—standard deviation taken as the measurement error.

**Table 5:** Air flow velocity in the door opening for fan 4 where: x—position of the measuring point in relation to the x-axis and, y—position of the measuring point in relation to the y-axis.

Distance of the positive pressure ventilator 4 from the door opening 1m The inclination angle of the positive pressure ventilator impeller relative to the ground 0°											
		x (mm)									
		91		273		455		637		819	
		AVG	SD	AVG	SD	AVG	SD	AVG	SD	AVG	SD
y (mm)	101.5	1.288	0.14	7.971	0.73	21.328	0.58	13.127	0.50	4.751	0.37
	304.5	3.066	0.53	19.445	0.59	23.2	0.31	23.276	0.78	6.336	0.60
	507.5	3.371	1.03	21.256	0.64	23.814	0.13	18.623	0.95	2.226	0.29
	710.5	0.743	0.15	4.72	0.56	10.096	0.55	3.119	0.62	0.99	0.16
	913.5	0.743	0.28	1.036	0.29	1.832	0.19	1.108	0.16	1.602	0.18
	1116.5	1.078	0.16	0.637	0.18	0.517	0.18	1.432	0.34	1.202	0.23
	1319.5	0.484	0.12	0.562	0.28	0.395	0.15	0.594	0.19	0.441	0.19
	1522.5	0.589	0.29	0.463	0.08	0.428	0.09	0.278	0.03	0.455	0.12
	1725.5	0.289	0.03	0.373	0.06	0.306	0.08	0.373	0.07	0.237	0.04
	1928.5	0.302	0.07	0.251	0.08	0.195	0.01	0.237	0.03	0.276	0.09

Distance of the positive pressure ventilator 4 from the door opening 1m The inclination angle of the positive pressure ventilator impeller relative to the ground 5°											
		x (mm)									
		91		273		455		637		819	
		AVG	SD	AVG	SD	AVG	SD	AVG	SD	AVG	SD
y (mm)	101.5	1.991	0.16	2.007	0.24	6.281	1.15	3.865	0.37	3.442	0.28
	304.5	1.772	0.18	10.576	1.17	25.335	0.68	13.619	0.79	5.013	0.39
	507.5	3.24	0.21	23.72	0.41	21.291	0.31	24.635	0.75	6.792	0.77
	710.5	1.268	0.48	19.221	1.12	23.598	0.42	14.852	1.39	1.648	0.33
	913.5	1.268	0.25	2.557	0.44	6.357	1.15	2.483	0.48	0.754	0.15
	1116.5	0.671	0.14	0.942	0.30	0.715	0.17	0.993	0.37	0.582	0.25
	1319.5	0.485	0.08	0.322	0.05	0.44	0.12	0.265	0.03	0.325	0.10
	1522.5	1.085	0.15	1.299	0.41	0.322	0.06	0.36	0.08	0.365	0.07
	1725.5	0.276	0.04	0.312	0.05	0.23	0.02	0.239	0.03	0.306	0.10
	1928.5	0.252	0.05	0.307	0.09	0.209	0.02	0.197	0.01	0.197	0.01

Distance of the positive pressure ventilator 4 from the door opening 1m The inclination angle of the positive pressure ventilator impeller relative to the ground 11°											
		x (mm)									
		91		273		455		637		819	
		AVG	SD	AVG	SD	AVG	SD	AVG	SD	AVG	SD
y (mm)	101.5	2.083	0.12	1.634	0.43	2.408	0.16	2.287	0.43	1.789	0.32
	304.5	1.124	0.28	4.739	1.74	9.642	0.93	5.773	0.65	2.875	0.72
	507.5	3.346	1.15	16.367	1.36	26.249	0.39	18.876	0.84	5.089	0.65
	710.5	3.499	0.98	23.759	0.72	22.357	0.35	23.196	0.31	5.861	1.21
	913.5	3.499	0.85	15.467	1.19	22.3	0.31	11.157	0.85	3.169	0.84
	1116.5	1.172	0.38	3.326	0.88	3.856	0.66	1.88	0.31	0.965	0.16
	1319.5	0.582	0.16	0.597	0.12	0.764	0.15	0.788	0.17	0.57	0.07
	1522.5	0.519	0.07	0.54	0.06	0.394	0.05	0.469	0.08	0.549	0.23
	1725.5	0.259	0.04	0.382	0.09	0.423	0.13	0.493	0.10	0.522	0.12
	1928.5	0.292	0.05	0.219	0.03	0.36	0.11	0.305	0.05	0.302	0.06

Distance of the positive pressure ventilator 4 from the door opening 1m The inclination angle of the positive pressure ventilator impeller relative to the ground 17°											
		x (mm)									
		91		273		455		637		819	
		AVG	SD	AVG	SD	AVG	SD	AVG	SD	AVG	SD
y (mm)	101.5	1.333	0.48	2.073	0.15	1.707	0.31	2.115	0.14	1.59	0.41
	304.5	1.446	0.20	1.051	0.19	1.552	0.23	2.083	0.50	1.661	0.20
	507.5	0.824	0.09	5.201	0.69	14.653	1.79	8.107	1.33	3.237	1.02
	710.5	5.579	0.43	17.504	1.18	25.872	0.61	22.043	1.18	7.502	1.05
	913.5	5.579	0.98	22.457	1.04	24.572	0.59	22.243	0.72	8.186	1.09
	1116.5	4.149	0.59	14.021	1.19	18.874	0.76	9.416	0.79	3.332	0.66
	1319.5	1.023	0.19	2.136	0.67	2.425	0.54	1.326	0.35	1.419	0.18
	1522.5	0.407	0.03	0.43	0.05	1.687	0.39	1.029	0.48	0.678	0.15
	1725.5	0.445	0.08	0.602	0.22	0.804	0.15	0.766	0.07	0.396	0.09
	1928.5	0.58	0.19	0.366	0.07	0.359	0.11	0.436	0.06	0.35	0.06

Distance of the positive pressure ventilator 4 from the door opening 3 m The inclination angle of the positive pressure ventilator impeller relative to the ground 0°											
		x (mm)									
		91		273		455		637		819	
		AVG	SD	AVG	SD	AVG	SD	AVG	SD	AVG	SD
y (mm)	101.5	7.377	0.71	12.154	0.76	15.42	0.70	16.335	0.72	13.874	0.58
	304.5	9.967	1.11	17.289	0.88	20.872	0.35	17.352	0.48	10.487	1.20
	507.5	10.023	1.38	17.897	0.41	17.825	0.60	13.01	0.76	7.179	0.62
	710.5	3.463	1.93	6.936	0.74	7.586	1.79	7.705	1.35	5.903	1.03
	913.5	3.463	1.46	2.1	0.58	1.79	0.49	2.367	0.66	1.903	0.19
	1116.5	1.968	0.26	1.544	0.50	1.266	0.20	1.634	0.27	1.687	0.17
	1319.5	2.089	0.31	1.364	0.38	1.681	0.24	1.195	0.22	1.546	0.35
	1522.5	0.902	0.45	0.756	0.29	0.505	0.13	0.573	0.24	1.094	0.50
	1725.5	0.848	0.20	0.703	0.20	1.051	0.49	0.76	0.47	0.538	0.22
	1928.5	0.46	0.07	0.592	0.19	0.422	0.19	0.524	0.18	0.325	0.10

Distance of the positive pressure ventilator 4 from the door opening 3 m The inclination angle of the positive pressure ventilator impeller relative to the ground 5°											
		x (mm)									
		91		273		455		637		819	
		AVG	SD	AVG	SD	AVG	SD	AVG	SD	AVG	SD
y (mm)	101.5	1.771	0.43	3.952	1.41	5.3	1.34	8.061	1.11	7.404	0.44
	304.5	3.782	0.96	8.603	1.41	10.603	1.34	11.258	0.58	8.372	1.03
	507.5	7.421	1.60	12.528	1.61	15.771	0.68	12.651	0.98	7.037	0.93
	710.5	9.635	0.91	18.706	0.65	19.912	0.61	12.888	2.01	5.054	1.30
	913.5	9.635	1.89	15.174	0.86	14.68	1.32	11.338	1.51	5.407	1.13
	1116.5	9.292	0.77	7.279	0.93	4.523	0.59	3.918	1.30	1.171	0.25
	1319.5	3.018	0.83	1.591	0.44	1.278	0.31	0.76	0.11	0.749	0.13
	1522.5	1.588	0.27	1.445	0.19	0.812	0.30	0.674	0.30	0.57	0.17
	1725.5	1.16	0.32	0.686	0.38	0.853	0.17	0.603	0.20	0.398	0.05
	1928.5	0.797	0.27	0.516	0.13	0.508	0.14	0.484	0.10	0.365	0.10

Distance of the positive pressure ventilator 4 from the door opening 3 m The inclination angle of the positive pressure ventilator impeller relative to the ground 11°											
		x (mm)									
		91		273		455		637		819	
		AVG	SD	AVG	SD	AVG	SD	AVG	SD	AVG	SD
y (mm)	101.5	1.488	0.36	1.221	0.31	1.934	0.26	2.201	0.27	1.718	0.46
	304.5	1.524	0.27	1.899	0.26	1.573	0.22	2.166	0.84	2.421	0.83
	507.5	1.902	0.32	3.721	0.94	5.643	1.31	5.897	1.36	7.572	2.70
	710.5	8.537	0.62	7.418	1.33	11.077	1.54	11.092	1.01	9.616	0.80
	913.5	8.537	1.20	13.687	0.92	19.03	0.79	15.586	0.79	11.121	0.88
	1116.5	9.762	1.05	17.072	0.59	19.31	0.89	13.81	1.68	8.013	0.60
	1319.5	8.334	1.21	12.798	0.86	14.542	1.06	9.956	0.83	5.908	1.05
	1522.5	7.534	0.74	7.907	0.97	4.814	0.78	5.147	1.06	3.46	0.98
	1725.5	3.493	0.70	2.684	1.12	2.297	0.64	2.327	0.81	2.069	0.43
	1928.5	0.681	0.13	0.847	0.20	0.635	0.19	0.733	0.21	1.11	0.24

Distance of the positive pressure ventilator 4 from the door opening 3 m The inclination angle of the positive pressure ventilator impeller relative to the ground 17°											
		x (mm)									
		91		273		455		637		819	
		AVG	SD	AVG	SD	AVG	SD	AVG	SD	AVG	SD
y (mm)	101.5	1.043	0.15	1.437	0.20	1.223	0.29	1.368	0.34	1.237	0.24
	304.5	1.345	0.17	1.775	0.39	0.73	0.18	1.367	0.28	1.495	0.37
	507.5	0.593	0.11	0.999	0.30	1.524	0.49	1.598	0.39	1.343	0.29
	710.5	1.624	0.09	0.961	0.25	0.968	0.31	1.62	0.43	1.813	0.51
	913.5	1.624	0.38	2.616	0.38	4.118	0.80	4.122	0.84	5.598	1.10
	1116.5	4.564	0.70	9.189	0.75	13.003	0.87	12.413	0.38	9.425	0.73
	1319.5	6.872	1.32	13.433	0.49	18.23	0.55	17.246	0.48	10.643	0.54
	1522.5	10.738	1.11	16.977	0.73	21.625	0.34	16.018	2.34	9.228	0.79
	1725.5	8.9	0.58	15.653	0.87	17.144	0.58	12.062	0.83	4.917	0.57
	1928.5	6.397	0.45	9.169	0.58	5.961	0.98	6.734	0.91	3.304	0.72

Distance of the positive pressure ventilator 4 from the door opening 4 m The inclination angle of the positive pressure ventilator impeller relative to the ground 0°											
		x (mm)									
		91		273		455		637		819	
		AVG	SD	AVG	SD	AVG	SD	AVG	SD	AVG	SD
y (mm)	101.5	7.792	0.56	11.152	1.17	15.302	0.89	14.722	0.46	14.983	0.79
	304.5	10.482	1.00	16.089	0.69	18.13	0.54	14.893	0.58	10.39	0.63
	507.5	11.949	0.71	16.119	0.58	15.534	0.55	11.362	0.81	7.887	1.46
	710.5	5.104	0.76	11.538	0.26	10.356	0.67	8.091	0.33	5.228	0.87
	913.5	5.104	1.28	5.687	1.04	6.287	1.07	4.291	0.62	2.338	0.35
	1116.5	2.776	0.51	2.849	0.94	2.101	0.52	1.52	0.37	1.287	0.21
	1319.5	1.336	0.32	1.404	0.14	1.313	0.21	1.389	0.34	1.424	0.52
	1522.5	0.9	0.38	1.485	0.36	0.68	0.09	0.842	0.16	1.632	0.29
	1725.5	0.653	0.16	1.773	0.60	1.034	0.32	1.473	0.34	0.795	0.17
	1928.5	0.99	0.36	0.537	0.11	1.087	0.17	0.628	0.16	0.527	0.11

Distance of the positive pressure ventilator 4 from the door opening 4 m The inclination angle of the positive pressure ventilator impeller relative to the ground 5°											
		x (mm)									
		91		273		455		637		819	
		AVG	SD	AVG	SD	AVG	SD	AVG	SD	AVG	SD
y (mm)	101.5	2.786	0.53	4.107	0.70	5.708	1.39	7.397	1.52	6.105	0.65
	304.5	5.617	1.00	7.528	1.50	9.556	1.02	8.273	1.01	7.93	0.50
	507.5	9.598	1.04	12.828	1.09	14.406	0.46	11.67	0.91	8.407	0.63
	710.5	13.022	1.12	16.681	0.48	14.976	0.99	11.477	1.23	8.224	1.30
	913.5	13.022	0.97	15.484	0.72	14.749	0.88	10.933	1.29	6.758	0.99
	1116.5	8.639	0.96	11.526	0.58	10.039	1.66	8.945	0.86	5.088	0.81
	1319.5	4.686	1.78	6.437	1.34	3.997	0.74	3.2	0.67	2.408	0.78
	1522.5	4.015	0.83	2.811	0.87	1.435	0.32	1.28	0.20	2.192	0.42
	1725.5	1.556	0.38	1.368	0.28	1.011	0.20	0.962	0.13	1.44	0.50
	1928.5	1.18	0.34	1.243	0.20	0.868	0.24	0.477	0.10	0.909	0.42

Distance of the positive pressure ventilator 4 from the door opening 4 m The inclination angle of the positive pressure ventilator impeller relative to the ground 11°											
		x (mm)									
		91		273		455		637		819	
		AVG	SD	AVG	SD	AVG	SD	AVG	SD	AVG	SD
y (mm)	101.5	1.279	0.17	1.656	0.49	1.403	0.51	1.177	0.30	2.061	0.32
	304.5	0.985	0.25	1.33	0.22	1.733	0.44	1.933	0.33	1.874	0.77
	507.5	1.83	0.65	2.238	0.50	2.339	0.68	3.79	1.08	4.767	0.78
	710.5	6.284	0.44	5.35	1.17	5.835	0.79	5.631	1.03	6.595	0.74
	913.5	6.284	0.75	8.63	0.77	10.192	1.09	12.685	1.00	10.001	0.62
	1116.5	7.003	1.02	12.766	1.04	16.143	0.67	14.884	0.43	11.102	0.76
	1319.5	8.18	0.61	14.923	0.80	16.409	1.17	12.96	0.55	8.44	1.17
	1522.5	8.649	0.87	13.438	0.65	14.895	0.71	10.843	0.68	7.608	0.56
	1725.5	6.472	0.99	8.962	1.33	9.671	0.72	5.409	1.09	3.948	0.88
	1928.5	3.495	0.85	2.034	0.47	2.34	0.64	2.673	0.61	2.914	0.61

Distance of the positive pressure ventilator 4 from the door opening 4 m The inclination angle of the positive pressure ventilator impeller relative to the ground 17°											
		x (mm)									
		91		273		455		637		819	
		AVG	SD	AVG	SD	AVG	SD	AVG	SD	AVG	SD
y (mm)	101.5	0.603	0.23	1.005	0.17	1.281	0.18	1.944	0.24	1.182	0.34
	304.5	0.602	0.18	0.562	0.10	1.148	0.21	1.894	0.33	1.469	0.48
	507.5	0.497	0.09	1.031	0.30	1.443	0.31	0.96	0.29	1.471	0.31
	710.5	0.762	0.30	0.584	0.10	0.738	0.16	1.092	0.20	1.377	0.30
	913.5	0.762	0.16	1.382	0.34	1.116	0.22	1.441	0.33	1.725	0.54
	1116.5	2.444	0.64	3.518	0.92	3.261	0.38	5.171	0.58	6.076	0.85
	1319.5	4.679	0.69	6.188	0.80	7.438	1.55	8.54	0.52	8.458	0.96
	1522.5	6.99	0.39	10.201	0.57	12.009	1.10	14.59	0.56	10.497	0.60
	1725.5	9.154	0.68	13.455	0.56	17.315	0.65	16.777	0.44	12.364	0.82
	1928.5	10.622	0.43	14.505	0.59	15.085	1.00	14.482	0.50	11.616	0.49

Distance of the positive pressure ventilator 4 from the door opening 5 m The inclination angle of the positive pressure ventilator impeller relative to the ground 0°											
		x (mm)									
		91		273		455		637		819	
		AVG	SD	AVG	SD	AVG	SD	AVG	SD	AVG	SD
y (mm)	101.5	8.284	0.56	12.24	0.61	13.674	0.43	14.203	0.43	13.129	0.55
	304.5	10.591	0.84	14.292	0.37	15.507	0.49	13.152	0.57	10.697	0.73
	507.5	10.038	0.83	13.55	0.53	14.052	0.37	10.556	0.99	8.692	0.64
	710.5	7.614	0.72	9.879	1.15	10.438	0.43	8.325	0.77	6.167	0.61
	913.5	7.614	0.88	7.619	0.71	5.727	0.68	5.626	0.64	4.549	0.43
	1116.5	3.825	0.67	3.818	0.58	3.942	0.57	2.338	0.35	2.246	0.45
	1319.5	1.406	0.19	2.544	0.52	1.699	0.46	1.605	0.27	1.309	0.23
	1522.5	0.936	0.22	1.295	0.16	1.154	0.13	1.318	0.17	1.125	0.20
	1725.5	0.737	0.22	0.886	0.12	0.779	0.10	0.905	0.26	1.368	0.45
	1928.5	0.981	0.34	1.946	0.33	0.985	0.21	0.642	0.07	0.587	0.19

Distance of the positive pressure ventilator 4 from the door opening 5 m The inclination angle of the positive pressure ventilator impeller relative to the ground 5°											
		x (mm)									
		91		273		455		637		819	
		AVG	SD	AVG	SD	AVG	SD	AVG	SD	AVG	SD
y (mm)	101.5	2.944	0.76	3.741	0.36	5.158	0.66	5.989	0.79	7.664	1.32
	304.5	4.953	1.06	6.595	0.84	8.497	0.69	8.602	0.73	8.601	0.84
	507.5	7.334	1.08	9.632	0.80	10.84	0.83	10.155	0.83	8.607	0.80
	710.5	11.388	0.36	12.049	0.80	12.939	0.71	11.021	0.97	7.946	0.79
	913.5	11.388	0.84	14.076	0.84	13.485	1.05	10.47	0.82	7.827	0.69
	1116.5	11.212	0.73	12.333	0.48	10.582	0.68	8.385	1.64	5.75	0.93
	1319.5	9.389	0.75	9.112	0.58	8.241	0.95	6.093	0.71	4.408	0.70
	1522.5	5.024	0.65	5.674	0.59	4.6	0.70	2.99	0.42	2.491	0.51
	1725.5	2.494	0.44	2.795	0.58	2.229	0.59	2.017	0.48	1.273	0.21
	1928.5	1.996	0.36	1.394	0.25	1.136	0.35	1.023	0.21	0.753	0.12

Distance of the positive pressure ventilator 4 from the door opening 5 m The inclination angle of the positive pressure ventilator impeller relative to the ground 11°											
		x (mm)									
		91		273		455		637		819	
		AVG	SD	AVG	SD	AVG	SD	AVG	SD	AVG	SD
y (mm)	101.5	0.753	0.15	0.693	0.10	1.766	0.49	1.897	0.54	1.518	0.20
	304.5	1.296	0.28	1.462	0.17	1.834	0.27	1.443	0.31	1.711	0.32
	507.5	1.215	0.21	1.324	0.24	1.329	0.36	1.723	0.62	1.894	0.79
	710.5	3.27	0.23	2.715	0.60	4.432	0.58	4.33	0.39	4.717	0.94
	913.5	3.27	0.35	5.124	0.81	6.552	0.41	6.116	0.72	7.463	0.55
	1116.5	7.102	0.66	7.827	0.73	9.272	1.05	10.857	0.47	9.234	0.74
	1319.5	7.396	0.86	10.546	0.68	13.967	0.57	13.354	0.54	10.452	0.59
	1522.5	9.545	0.67	12.265	0.45	14.942	0.56	14.766	0.23	9.86	0.67
	1725.5	7.854	0.81	12.081	0.86	12.766	0.69	11.527	0.54	8.048	0.56
	1928.5	7.626	1.11	8.841	0.93	8.74	1.00	8.76	0.74	6.215	1.03

Distance of the positive pressure ventilator 4 from the door opening 5 m The inclination angle of the positive pressure ventilator impeller relative to the ground 17°											
		x (mm)									
		91		273		455		637		819	
		AVG	SD	AVG	SD	AVG	SD	AVG	SD	AVG	SD
y (mm)	101.5	0.906	0.19	0.976	0.15	1.587	0.34	1.309	0.31	1.862	0.29
	304.5	0.717	0.15	0.855	0.36	2.025	0.18	1.868	0.25	1.899	0.28
	507.5	1.493	0.42	1.496	0.35	1.301	0.27	1.125	0.43	1.225	0.34
	710.5	0.943	0.21	1.073	0.30	0.789	0.18	1.053	0.24	1.073	0.23
	913.5	0.943	0.25	1.065	0.34	1.062	0.16	1.384	0.53	2.143	0.52
	1116.5	1.639	0.46	1.474	0.43	1.307	0.41	1.524	0.39	3.331	0.78
	1319.5	2.241	0.57	2.459	0.88	2.916	0.64	5.323	0.87	5.886	0.60
	1522.5	7.066	0.61	7.066	0.65	6.828	0.51	7.165	0.50	8.461	0.73
	1725.5	8.124	0.38	9.403	0.67	11.237	0.51	10.741	0.34	10.677	0.74
	1928.5	10.573	1.34	12.697	1.06	14.458	0.77	13.952	0.51	11.682	0.35

Distance of the positive pressure ventilator 4 from the door opening 7 m The inclination angle of the positive pressure ventilator impeller relative to the ground 0°											
		x (mm)									
		91		273		455		637		819	
		AVG	SD	AVG	SD	AVG	SD	AVG	SD	AVG	SD
y (mm)	101.5	8.365	0.50	11.304	0.59	12.455	0.51	12.134	0.37	11.052	0.35
	304.5	10.122	0.52	11.728	0.48	11.861	0.49	10.709	0.69	10.476	0.37
	507.5	9.579	0.56	10.522	0.53	10.365	0.59	9.474	0.36	8.572	0.56
	710.5	8.726	0.65	8.855	0.44	8.252	0.65	7.847	0.78	7.993	0.50
	913.5	8.726	0.57	7.95	0.47	6.641	0.38	5.069	0.42	3.064	0.57
	1116.5	3.432	0.52	4.992	0.39	4.279	0.46	3.7	0.76	3.414	0.79
	1319.5	1.904	0.52	3.505	0.25	3.262	0.47	2.278	0.54	1.568	0.34
	1522.5	3.447	0.84	2.149	0.37	1.645	0.38	1.623	0.35	1.861	0.48
	1725.5	0.777	0.15	1.536	0.26	1.448	0.27	1.326	0.15	1.418	0.17
	1928.5	1.159	0.14	1.025	0.14	0.755	0.21	0.805	0.15	1.362	0.19

Distance of the positive pressure ventilator 4 from the door opening 7 m The inclination angle of the positive pressure ventilator impeller relative to the ground 5°											
		x (mm)									
		91		273		455		637		819	
		AVG	SD	AVG	SD	AVG	SD	AVG	SD	AVG	SD
y (mm)	101.5	3.687	0.96	4.621	0.82	5.701	0.88	7.268	0.85	7.6	0.70
	304.5	5.524	0.68	6.164	0.57	8.035	0.51	8.187	0.64	8.405	0.51
	507.5	5.867	0.76	7.615	0.42	9.006	0.59	8.041	0.54	8.276	0.46
	710.5	7.882	0.50	8.83	0.67	8.781	0.68	8.469	0.68	8.47	0.61
	913.5	7.882	0.95	9.668	0.29	9.093	0.63	8.083	0.67	8.046	0.62
	1116.5	9.148	0.45	10.3	0.65	8.98	0.78	7.646	0.99	6.829	0.46
	1319.5	8.046	0.85	7.901	0.56	8.009	0.67	6.124	0.70	5.358	0.61
	1522.5	6.086	0.72	8.378	0.43	5.502	0.56	5.47	0.64	2.791	0.38
	1725.5	4.082	0.50	5.822	0.67	4.179	0.59	4.573	0.50	2.85	0.99
	1928.5	2.495	0.75	3.423	0.47	3.081	0.44	3.045	0.37	2.271	0.52

Distance of the positive pressure ventilator 4 from the door opening 7 m The inclination angle of the positive pressure ventilator impeller relative to the ground 11°											
		x (mm)									
		91		273		455		637		819	
		AVG	SD	AVG	SD	AVG	SD	AVG	SD	AVG	SD
y (mm)	101.5	0.996	0.26	1.363	0.40	2.082	0.42	1.56	0.63	1.227	0.31
	304.5	1.269	0.18	1.328	0.28	1.145	0.24	1.175	0.20	1.156	0.24
	507.5	1.272	0.31	1.056	0.14	0.88	0.19	1.009	0.22	1.468	0.54
	710.5	2.911	0.21	1.305	0.48	1.799	0.40	1.801	0.44	3.267	0.56
	913.5	2.911	0.84	3.361	0.52	2.683	0.51	4.528	0.69	4.337	0.73
	1116.5	4.337	0.57	4.167	0.68	4.897	0.69	5.077	0.52	5.325	0.48
	1319.5	5.46	0.36	6.143	0.53	6.453	0.75	6.771	0.57	6.92	0.25
	1522.5	7.343	0.66	8.469	0.64	8.036	0.42	8.234	0.47	7.329	0.79
	1725.5	8.125	0.50	9.347	0.50	9.682	0.48	9.302	0.38	8.263	0.45
	1928.5	9.317	0.55	9.847	0.63	9.836	0.56	9.879	0.55	9.334	0.41

Distance of the positive pressure ventilator 4 from the door opening 7 m The inclination angle of the positive pressure ventilator impeller relative to the ground 17°											
		x (mm)									
		91		273		455		637		819	
		AVG	SD	AVG	SD	AVG	SD	AVG	SD	AVG	SD
y (mm)	101.5	1.513	0.24	1.554	0.27	1.634	0.40	1.96	0.50	1.439	0.32
	304.5	1.586	0.19	2.436	0.11	2.444	0.50	1.65	0.53	1.116	0.37
	507.5	1.426	0.22	1.215	0.30	2.218	0.60	2.415	0.27	1.421	0.30
	710.5	0.709	0.26	0.757	0.23	0.771	0.19	0.919	0.22	1.208	0.33
	913.5	0.709	0.15	0.896	0.23	1.207	0.41	0.992	0.23	1.063	0.26
	1116.5	1.102	0.38	1.048	0.31	0.906	0.23	0.799	0.17	0.959	0.21
	1319.5	1.212	0.28	1.776	0.41	1.282	0.32	2.235	0.45	1.145	0.40
	1522.5	2.108	0.42	2.668	0.37	2.571	0.42	3.001	0.63	2.793	0.55
	1725.5	3.407	0.71	4.043	0.57	3.919	0.63	4.072	0.47	4.001	0.87
	1928.5	3.09	0.48	1.992	0.32	1.726	0.43	2.41	0.67	4.102	0.96

AVG—arithmetic mean of the flow rate, SD—standard deviation taken as the measurement error.

*V*—average value of the flow velocity of the air stream generated by the positive pressure ventilator, *S*—measuring area of the door opening (measurement plane).

In the analysis of the measurement error (for the air flow velocity tests at selected measuring points), the arithmetic mean was used as an estimator of the value. The standard deviation of the arithmetic mean was adopted as the error of the estimator. Whereas the main test results provided average values of air flow rate from 50 trials (*N* = 50), for which confidence intervals were determined at a confidence level of 95% (*p* = 0.05). Significant statistical differences were analyzed using Student’s t-test. The tests were carried out in a research hall with a cubage of 1500 m^3^. During the test, the door to the hall was open. While carrying out the research, environmental conditions were monitored, which were as follows: temperature (21 ± 2 °C), humidity (41 ± 3%), pressure (1003 ± 10 hPa). The experiment was carried out in conditions when the speed value was below the wind ± 0.3 m/s. All obstacles were located away from the test stand (positive pressure ventilator—air flow path—test equipment), at a distance of not less than 10 × D (D—the diameter of the impeller of the largest tested fan).

Describing the research in question for the positive pressure ventilator in the presented setup, some of its limitations should also be mentioned. The experiment was carried out using a mock-up simulating a door opening, on which a measurement plane was located, without the cubic capacity (building structure) behind the door opening. Bearing in mind the above, it should be noted that the implemented method does not allow for the assessment of the amount of air flow in the conditions of back-pressure, which is present in the building and will be variable depending on its construction and the conditions prevailing in it. The measured volumetric flow is the upper limit of the volumetric flow that can be generated by a positive pressure ventilator (corresponding to infinitely small flow resistances and thus no back pressure). In addition, it should also be pointed out that the presented conclusions regarding the parameters of the effective positioning of mobile fans may not be the same for other units available on the market.

**Figure 3:**
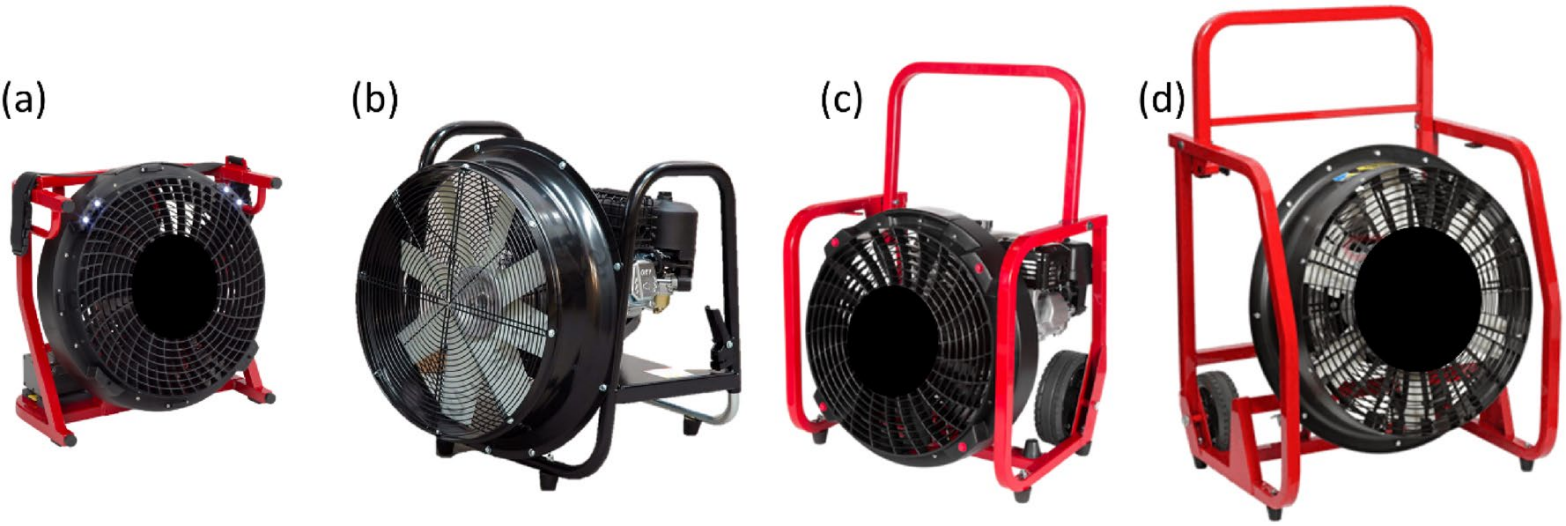
Tested positive pressure ventilators, where: fan (**a**) marked as 1, (**b**)—2, (**c**)—3, (**d**)—4.

Four positive pressure ventilators, popularly used in rescue operations, were used for the study (Fig. 3). Among other parameters, the fans are characterized by different ranges of drive power (from 0.6 to 6.3 kW). The description of the fan parameters is presented in Table 1.

## Results

**Figure 4:**
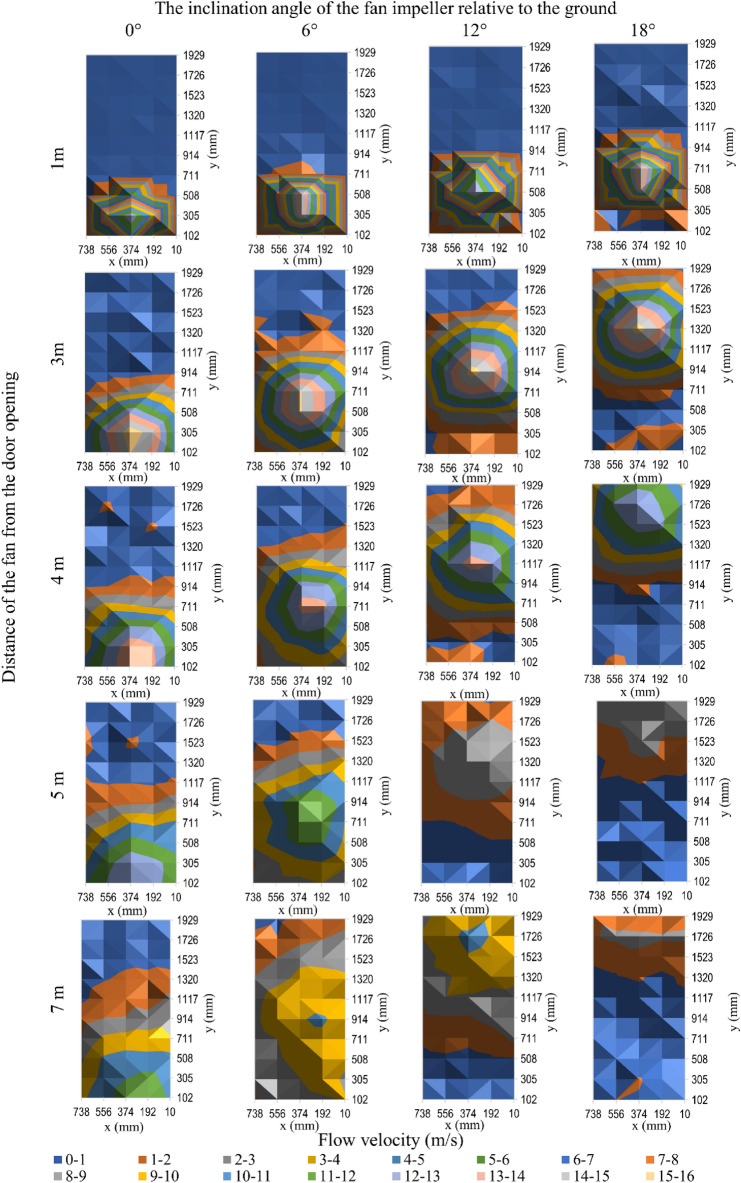
The velocity of air flow in the door opening for fan 1.

**Figure 5:**
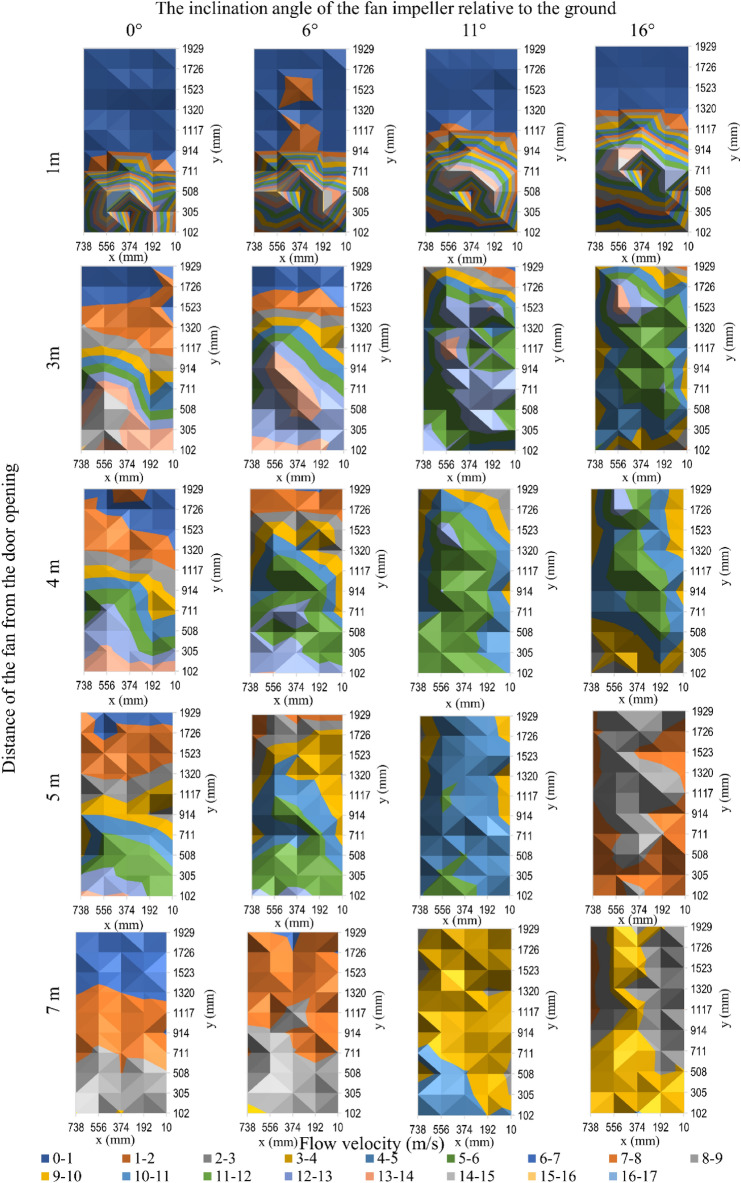
The velocity of air flow in the door opening for fan 2.

**Figure 6:**
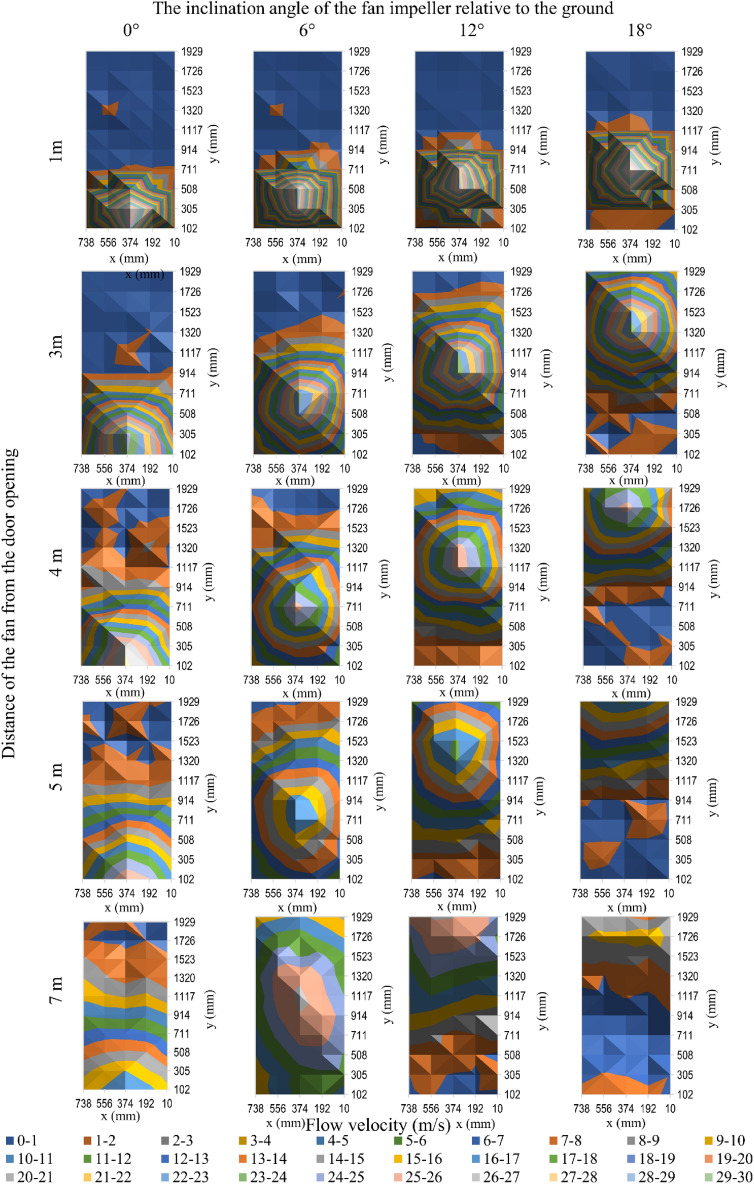
The velocity of air flow in the door opening for fan 3.

**Figure 7:**
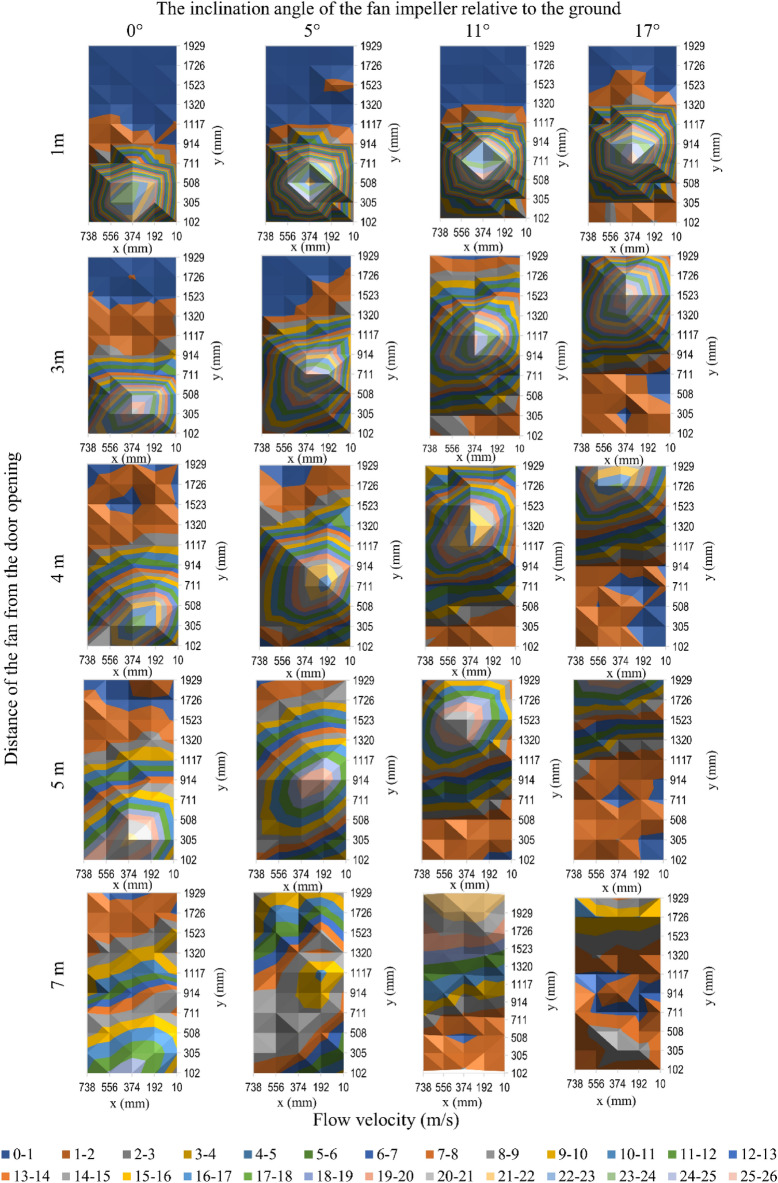
The velocity of air flow in the door opening for fan 4.

The results of testing the characteristics of the flow velocity profile on the surface of the door opening for the tested fans (taking into account unit positioning parameters, i.e., the distance and impeller inclination angle) are shown in Fig. 4 for the positive pressure ventilator 1, Fig. 5 for fan 2, Fig. 6 for fan 3 and Fig. 7 for mobile fan 4. Due to a large amount of information in the drawings, information on the accuracy of the measurement was not marked on them, therefore, Tables 2, 3, 4 and 5 present the details of the average results of the flow velocity and errors from the measurements in the doorway.

**Figure 8:**
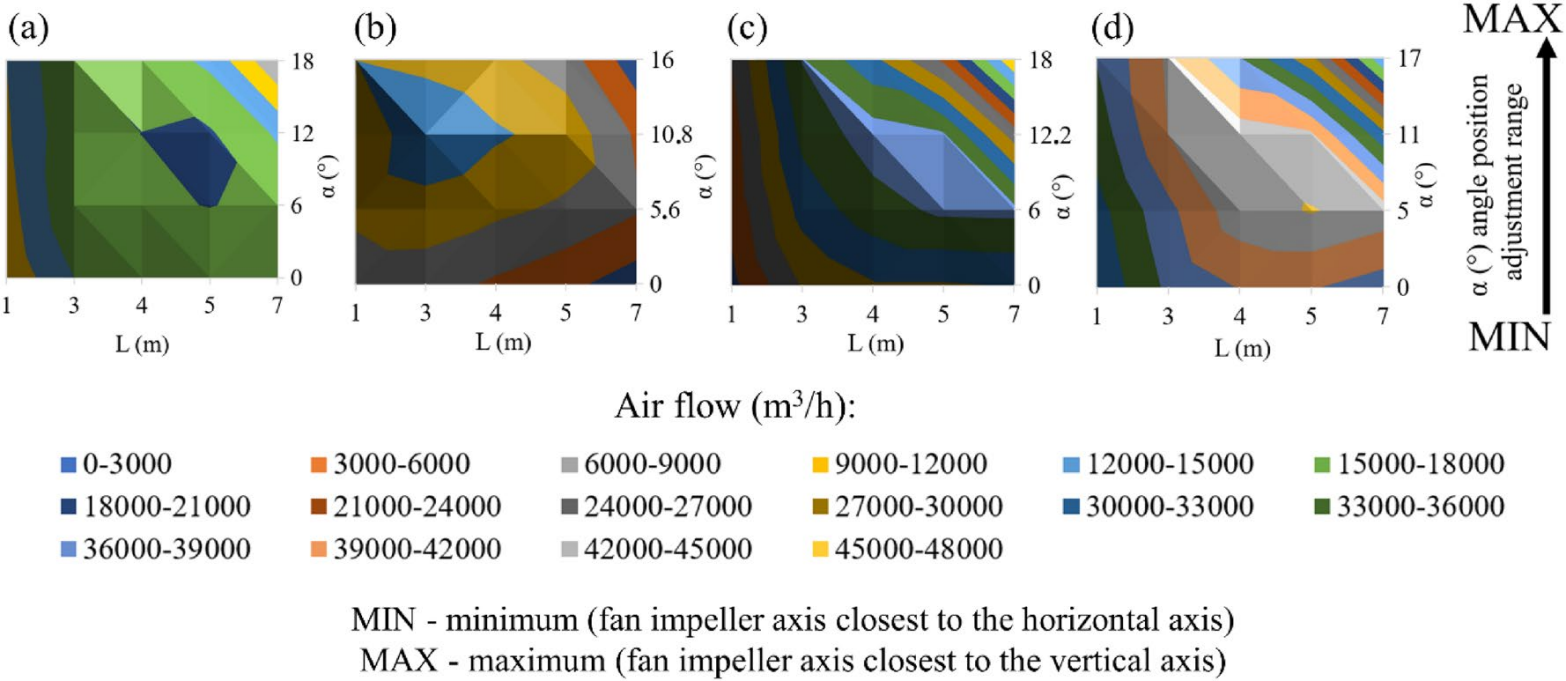
The influence of the distance of the positive pressure ventilator position from the door opening and the impeller inclination angle, where (**a**) ventilator 1, (**b**)—2, (**c**)—3, (**d**)—4, L—distance of the fan from the door opening, α—the angle of inclination of the impeller axis from the ground.

**Figure 9:**
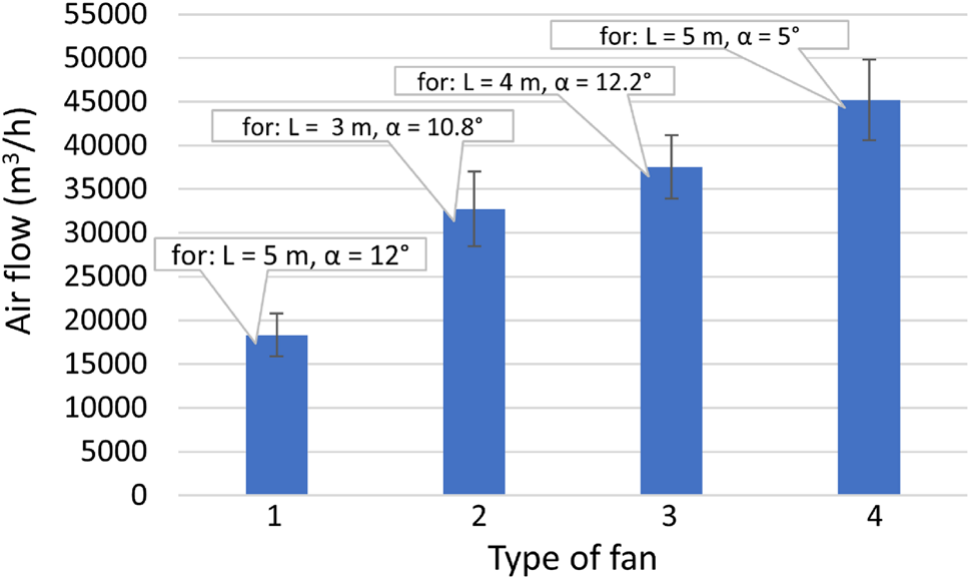
Maximum values of the volumetric flow rate with an indication of positive pressure ventilator settings, where: L—the distance of the mobile fan from the door opening, α—the angle of inclination of the impeller axis from the ground.

It can be seen from the flow rate characteristics in Figs. 4, 5, 6 and 7 that when the main flow is concentrated too close to the lower or upper edge of the door opening, it is less than the maximum obtainable value. The highest value of the flow velocity can be obtained when the airflow is aimed toward the centre area of the door opening. When analyzing the issues regarding the positioning of the positive pressure ventilator, it should be pointed out that if the rotor axis is tilted to the extreme position (up to 16° or 18°), the air stream does not completely hit the door opening or it loses momentum as a result of friction with the ground surface. Similar observations are noted when the axis of the fan impeller is set parallel to the ground. The loss of mass value of the flowing air stream as a result of a collision with the outer surface of the wall around the inlet opening was also shown by Cimolino et al.^17^, who tested the “cone” ventilation technique—a method that directs the flow so that the stream covers the entire opening. This technique was also described by Kaczmarzyk et al.^6^. Analyzing the issues related to the velocity of the air stream measured in the door opening, it was noted that Alonso et al.^18^ showed that the air flow through the door opening without active ventilation is concentrated in the lower part of the door opening (air outlet) at a speed of about 0.8 m/s, while the air inlet is in the upper part. Kerber and Walton ^19^, during their tests with the use of a mobile fan positioned at a distance of 3.05 m, evenly in the axis of the door opening, obtained a maximum value of air velocity of 6 m/s. In turn, the research of the authors of the article showed that the maximum flow velocity in the door opening during fan support may be equal to approximately 28 m/s. In further analysis, the results of the flow velocity were converted following Eq. (1), determining the volumetric air flow rate. The influence of the fan distance from the door opening and the impeller inclination angle are shown in Fig. 8. Due to the legibility of the drawing, no measurement error has been marked on it, hence the results of average values and measurement errors are presented in Table 6. On the other hand, the maximum values of the flow rate, with the indication of the fan settings, are shown in Fig. 9. The highest flow rates were achieved for the highest flow velocities. Depending on the power of the drive unit, the maximum flow rate ranged from approximately 18,304 ± 2460 m^3^/h (for a 0.6 kW fan) to approximately 45,189 ± 4619 m^3^/h (for a 6.3 kW fan). Lambert and Merci^20^ studied similar positive pressure ventilators, which are used in rescue operations. The indicated flow rates were respectively 30,800 m^3^/h for fans with a combustion engine and 30,000 m^3^/h for fans with an electric drive. Garcia et al.^21^ also indicated that fans used for rescue operations should generate a volumetric flow in the range of 25,485–33,980 m^3^/h. On the other hand, the mobile fan used by Kerber and Walton^19^ and Kerber^22^ had a capacity of 23,900 m^3^/h, which is consistent with the authors’ results. The distances from the door opening and the angles of the impeller axis, at which the highest values of the flow rate were obtained, fall within the range of 3–5 m, while the angles of the impeller axis to the ground range from 5° to 12°. Most often, the third position of the impeller axis (from the ground) proved to be more favourable than the other positions from among the four recommended by the manufacturer (12°). One of the tested mobile fans obtained the best results in the second position (5°). On the other hand, the first position was not favourable in any of the attempts. Therefore, when pumping air into rooms with a ground surface parallel to the fan base, it is recommended to change the position of the fan impeller axis. So far, changes in the position of the fan impeller axis were mainly recommended during ventilation in staircases, where a change of the air stream direction minimizes the loss of air momentum on obstacles located inside the building—e.g., non-standard staircase structures^23^. When analysing these results, it should be noted that the flow analysis is conducted through the door opening under conditions without back pressure (which may be present in the structure of the facility). The generated volume flow, pumped inside the object, is influenced by the pressure inside and the obstacles on the gas exchange path^6^. With regard to multi-storey buildings, Paninder et al.^24^ showed that the value of the flow rate is also influenced by the pressure difference at different levels of the staircase.

**Table 6:** The influence of the distance of fan location from the door opening and the angle of the impeller inclination on air flow rate for fans 1, 2, 3, and 4, where AVG—arithmetic mean flow rate (m3/h) and confidence interval (p = 0.05).

Positive pressure ventilator 1									
		The tilt angle of the positive pressure ventilator impeller axis with respect to the ground							
		0°		6°		12°		18°	
		AVG	p = 0.05	AVG	p = 0.05	AVG	p = 0.05	AVG	p = 0.05
Distance of the fan from the door opening	1 m	9660	613	10,764	811	11,547	1009	12,007	1180
	3 m	15,016	1839	17,587	2701	17,313	2231	17,966	2319
	4 m	15,620	1906	17,683	2547	18,013	2791	16,940	2146
	5 m	16,353	2004	18,058	2500	18,304	2460	14,322	1946
	7 m	15,192	2072	17,553	2726	14,550	2129	6185	1368

Positive pressure ventilator 2									
		The tilt angle of the positive pressure ventilator impeller axis with respect to the ground							
		0°		6°		11°		16°	
		AVG	p = 0.05	AVG	p = 0.05	AVG	p = 0.05	AVG	p = 0.05
Distance of the fan from the door opening	1 m	25,536	3150	27,597	3919	26,955	4304	29,943	4989
	3 m	25,426	2481	28,759	2958	32,744	4287	29,080	4350
	4 m	23,513	2337	27,671	2996	30,411	3391	27,816	3335
	5 m	21,764	2495	26,457	2770	28,901	2904	25,464	3753
	7 m	19,422	2324	24,360	2888	23,592	2968	19,122	3302

Positive pressure ventilator 3									
		The tilt angle of the positive pressure ventilator impeller axis with respect to the ground							
		0°		6°		12°		18°	
		AVG	p = 0.05	AVG	p = 0.05	AVG	p = 0.05	AVG	p = 0.05
Distance of the fan from the door opening	1 m	20,123	1 404	21,722	1 643	22,413	1 492	24,039	2 060
	3 m	27,230	2 492	31,116	3 258	33,808	3 796	36,147	3 555
	4 m	29,705	2 973	34,712	4 207	37,534	3 624	30,686	2 939
	5 m	29,695	2 686	36,629	4 023	36,249	3 538	23,310	2 691
	7 m	30,156	2 938	36,754	3 720	25,591	2 993	9045	1 955

Positive pressure ventilator 4									
		The tilt angle of the positive pressure ventilator impeller axis with respect to the ground							
		0°		5°		11°		17°	
		AVG	p = 0.05	AVG	p = 0.05	AVG	p = 0.05	AVG	p = 0.05
Distance of the fan from the door opening	1 m	30,494	1902	31,794	2092	34,249	2718	36,161	3255
	3 m	36,622	3755	38,212	4780	42,022	5212	42,483	3718
	4 m	39,158	3528	43,867	5133	43,531	4707	36,027	3122
	5 m	39,215	3144	45,189	4618	42,340	3745	27,664	2996
	7 m	37,547	2924	43,600	1180	30,782	3056	12,398	812

**Figure 10:**
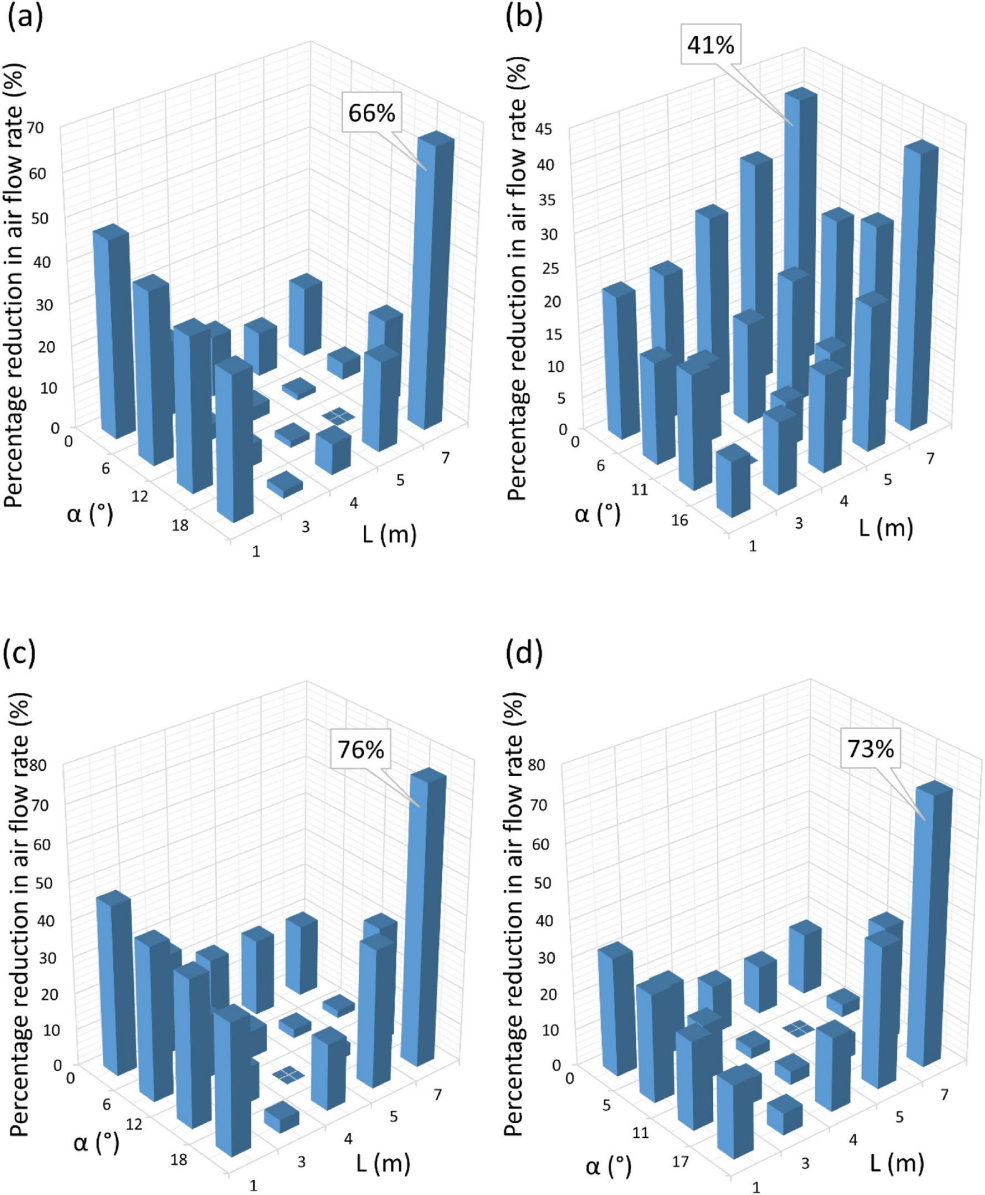
Percentage reduction of air flow depending on positive pressure ventilator setting, where: (**a**)—1, (**b**)—2, (**c**)—3, (**d**)—4, L—distance of the fan from the door opening, α—angle of inclination of the impeller axis from the ground.

The effectiveness of a rescue operation may depend on correct fan settings, therefore the analysis of the influence of the percentage reduction of the flow rate depending on mobile fan settings was performed (Fig. 10). The analysis adopted the result of the highest value of flow rate *Q*_*max*_ efficiency as a reference value for the selected fan, according to Eq. (2). Tests in the variable ranges of the distance from the door opening and the angle of inclination of the impeller axis have shown that improper fan settings may result in a maximum reduction of air flow through the door opening from 41 to 76%, depending on the fan type. Rejecting the results for the two most unfavourable distances and tilt angles of the impeller axis (i.e., extreme maximum and minimum positions) in the analysis, the greatest reduction of the flow rate ranges from 5 to 19% depending on the fan type. The differences in the volume of air flow are related to the change in the flow direction (the collision with an obstacle in the form of a door opening frame) and the quality of the generated air stream^7^. According to Cimolino et al.^17^ changing the angle of the blown air can increase the flow rate by up to 30%. Positive pressure ventilators producing an air stream with a lower degree of turbulence (e.g. having flow rectifiers), do not lose their momentum intensively (deceleration reduction), are able to blow a stably directed stream over longer distances (even 5–7 m)^28,29^. Positive pressure ventilators characterized by greater flow turbulence, e.g. conventional fan, (if set at distances above 1 m) may work with lower efficiency—the stream generated by the ventilator will begin to slow down as a result of adding additional air from the environment. Such a jet can also change the flow direction and asymmetrically flow onto the surface of the inlet opening^28,29^.

2 $$pr = \frac{{Q_{max} - Q}}{{Q_{max} }} \cdot 100\%$$

where:

*pr*—percentage reduction in air flow rate (%), ${Q}_{max}$—the value of the highest efficiency of the flow stream for the selected fan (m^3^/h), $Q$—value other than the highest flow rate efficiency for the selected fan (m^3^/h).

**Figure 11:**
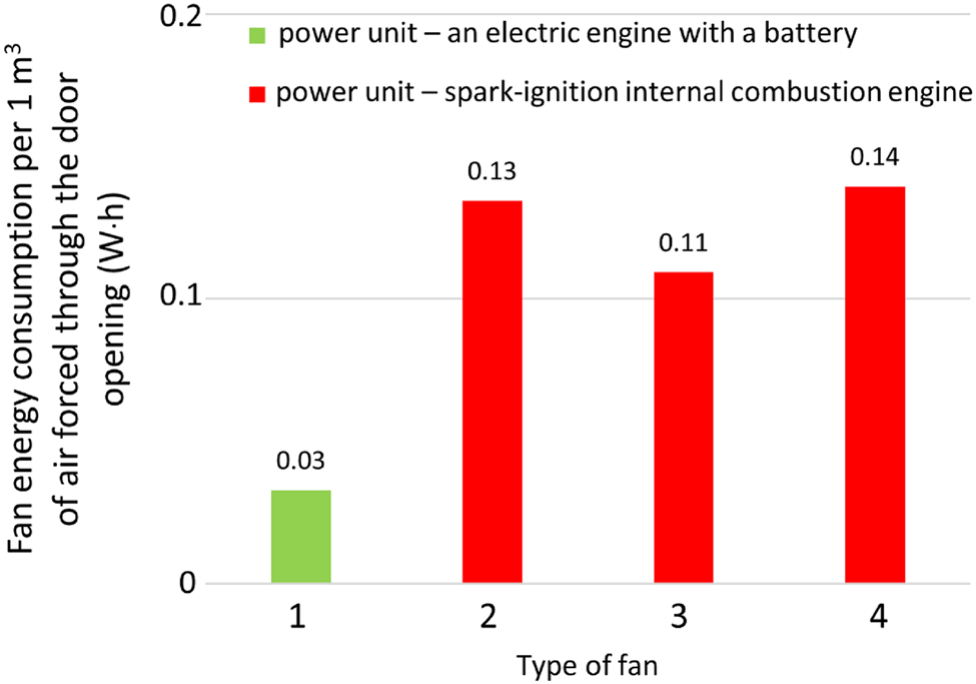
Fan energy consumption per 1 m^3^ of air blown through the door opening at the highest volumetric flow rate.

According to the results of the research by Kaczmarzyk et al. in 2022, Warguła et al. in 2023 and Warguła and Kaczmarzyk in 2022, it can be observed that mobile positive pressure ventilators run at maximum power^8,25,26^. By comparing the results of the maximum power of the drive units (Table 1) and the values of the maximum air flow rate generated by the mobile fan (Fig. 8), it is possible to determine the amount of energy consumed by the fan, expressed in Watt-hours (W h), per 1 m^3^ of air blown through the door opening according to Eq. (3). The value of the energy consumed per 1 m^3^ is shown in Fig. 11. It can be noted that the electric mobile fan is characterized by the lowest power (0.6 kW), the lowest maximum flow rate (18,304 ± 2460 m^3^/h) and the lowest energy consumption 0.03 W h. The remaining positive pressure ventilators (driven by combustion engines) were characterized by average energy consumption of about 0.13 ± 0.02 W h for the purpose of blowing 1 m^3^ of air through the door opening. It can be observed that mobile fans with combustion drives are characterized by 76% lower energy consumption. The efficiency of the combustion engine does not affect the value of this result because the value of the assumed power corresponds to the power on the drive shaft (output)^8,27^. The design of the fan impellers was also similar and of the same type. This can be influenced by the rotational speed of the fan impeller. Combustion engines, where fan impellers are mounted on the drive shaft, operate at a speed of about 3500 rpm^8^. The rotational speed of the electric motor shaft was 2790 rpm. However, the analysis of this issue and its confirmation requires further research.

3 $$e_{{1m^{3} }} = \frac{{Q_{{1m^{3} }} \cdot e_{1h work} }}{{fr_{max} }}$$

where:

${e}_{{1m}^{3}}$—energy consumption per 1 m^3^ of air pumped through the door opening (W h), ${e}_{1h work}$—energy consumption during 1 h of fan operation (W h), ${Q}_{{1m}^{3}}$—the value of 1 m^3^ of the flow stream for the selected fan (m^3^/h).

Based on the conducted research, it can be observed that the determination of the general guidelines for the setting of fans may be imprecise and unfavourable for their effective operation. Positive pressure ventilators, used for the tests, did not have information plates indicating the optimal settings of the mobile unit. The implementation of such instructions could contribute to increasing the effectiveness of the ventilation provided. Lambert et al. in 2018 studied the impact of fan settings for smoke extraction of subway stations, aiming at the critical speed in the tunnel of 2.37 m/s. Further work should be carried out on determining such parameters for smoke extraction of other buildings^30^.

## Conclusion

The correct setting of the positive pressure ventilator affects the value of the volumetric air flow blown through the door opening. The basic parameters that can be adjusted during rescue operations are the distance of the mobile fan from the door opening and the angle of impeller tilt in relation to the ground. The settings of these two parameters can significantly affect the air flow rate. For the four tested pressure ventilators (Table 1), commonly used in rescue operations (power from 0.6 to 6.3 kW), the generated value of the flow rate ranges from approximately 18,304 ± 2460 m^3^/h (for a 0.6 kW fan) to approximately 45,189 ± 4619 m^3^/h (for a 6.3 kW fan). The most favourable values of air flow rate are obtained at a distance in the range of 3–5 m from the door opening and settings of the impeller axis angle to the ground in the range of 5°–12°. When using these settings, the air stream flows in the centre of the door opening. It can be noted that when the positive pressure ventilator is set in a position ensuring flow in the central part of the door opening, it ensures the best flow velocity parameters. The tests have shown that even when air is pumped through the door opening into a room (with a surface parallel to the surface on which the fan is placed), it is advantageous to set the angle of inclination of the impeller axis from the ground to the second or third of four (from 5° to 12°) available positions, according to the recommended manufacturers’ settings. Taking the result of the highest efficiency of the flow rate as a reference value for the selected fan (during tests in the range of 1–7 m and within the range of the tested impeller axis inclination angles relative to the ground of 0°–18°), it has been found that imprecise setting may result in a reduction of the flow rate in relation to most favourable results ranging from 41 to 76% depending on the fan type. However, after rejecting the results from the most unfavourable ranges (extreme values of distances and angles), i.e., in the range of 3–5 m and the angle of inclination of the impeller axis from about 5° to 12°, the maximum reduction of the flow rate falls within the range of 5% to 19% depending on the fan type. The value of energy consumed per 1 m^3^ of air pumped by the tested fans ranges from 0.03 to 0.14 W h, depending on the fan type. The research expanded the state of knowledge about the ability to pump air with the use of mobile positive pressure fans in rescue operations, depending on the settings (the distance from the door opening and the angle of inclination of the impeller axis relative to the ground). The energy consumption of these processes was also determined. It should also be noted that the tests were conducted in an open space in one room, where the door opening was an obstacle, which may be a certain limitation in the analysis of the results. The developed test method does not allow for the assessment of the volumetric air flow rate, taking into account the pressure that may occur inside the ventilated volume of the building as a result of, e.g., an open window and the presence of wind flowing onto its surface. Research has shown that defining general guidelines for fan settings can be imprecise. Therefore, work should be carried out on the development of an information plate placed on the fan, suggesting favourable settings for selected rescue action scenarios and fan operating conditions.

## Data Availability

All data generated or analysed during this study are included in this published article.
